# Immune cell subset profiling and metabolic dysregulation define the divergent immune microenvironments in HIV immunological non‐responders

**DOI:** 10.1002/ctm2.70498

**Published:** 2025-10-13

**Authors:** Qingfei Chu, Ningye Fang, Huanhuan Chen, Abdur Rashid, Xia Luo, Jianjun Li, Kang Li

**Affiliations:** ^1^ Department of Infectious Diseases The Second Affiliated Hospital Zhejiang University School of Medicine Hangzhou China; ^2^ National Key Laboratory of Intelligent Tracking and Forecasting for Infectious Diseases Chinese Center for Disease Control and Prevention Beijing China; ^3^ Guangxi Key Laboratory of Major Infectious Disease Prevention Control and Biosafety Emergency Response Guangxi Center for Disease Control and Prevention Nanning China; ^4^ Division of Infectious Disease & International Medicine Morsani College of Medicine University of South Florida Tampa Florida USA; ^5^ Guangxi Key Laboratory of AIDS Prevention and Treatment School of Public Health Guangxi Medical University Nanning China; ^6^ Department of Microbiology School of Basic Medicine Guangxi Medical University Nanning China

**Keywords:** acquired immunodeficiency syndrome, hub genes, immune cell infiltration, immunological non‐responders, immunological responders, therapeutic targets

## Abstract

**Background:**

A subset of people living with HIV (PLWH) exhibit poor immune recovery despite effective antiretroviral therapy (ART), remaining at risk of disease progression. The immunometabolic mechanisms underlying this immunological non‐response remain unclear.

**Methods:**

We integrated transcriptomic and immunophenotypic approaches to characterise immune differences between immunological responders (IRs) and non‐responders (INRs). Public datasets were analysed to identify differentially expressed genes (DEGs), followed by enrichment analysis, predictive modelling, immune infiltration assessment, and regulatory network construction. In parallel, flow cytometry was performed to assess T and B cell subsets in an independent cohort including IRs, INRs, treatment‐naïve patients (TNPs), and healthy controls (HCs).

**Results:**

DEGs between IRs and INRs were enriched in mitochondrial and ribosomal pathways. INRs showed reduced Th1, Th17, and Tfh cells, alongside increased markers of immune activation and exhaustion. Predictive modelling identified five hub genes (*ATP5O, PIGY, UQCRQ, COX7C*, and *BLVRB*) associated with immune recovery, and clustering based on their expression defined two transcriptionally distinct subtypes. Flow cytometry further confirmed that INRs exhibited diminished CD4⁺ T cell counts, increased PD‐1⁺ and HLA‐DR⁺ expression, and reduced resting memory B cells, reflecting persistent immune dysfunction.

**Conclusions:**

This study underscores the pivotal role of immunometabolic dysregulation in shaping heterogeneous immune responses to ART. By integrating computational and experimental data, we identified key biomarkers and regulatory pathways associated with immune recovery. Our findings highlight the central influence of metabolic processes on immune restoration outcomes and propose personalised metabolic interventions as a promising strategy to enhance therapeutic efficacy in HIV‐infected individuals.

## Graphical abstract

Integrative transcriptomic and immunophenotypic profiling reveals that immunological non‐responders to ART display mitochondrial and ribosomal dysregulation, reduced Th1/Th17/Tfh responses, and impaired B cell memory. Five hub genes (*ATP5O, PIGY, UQCRQ, COX7C, BLVRB*) emerge as biomarkers, highlighting immunometabolic pathways as targets to enhance immune recovery in HIV.

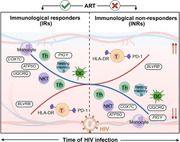

## 1 INTRODUCTION

Acquired immunodeficiency syndrome (AIDS), a serious and potentially life‐threatening condition, is caused by human immunodeficiency virus (HIV).^1^ More than four decades after the first case of HIV was identified in 1981, effective vaccines and curative treatments remain unavailable.^2^, ^3^ The advent of antiretroviral therapy (ART) has transformed AIDS from a fatal disease into a manageable chronic condition. ART remains the cornerstone of treatment and maintenance for people living with HIV (PLWH).^4^, ^5^ According to the 2024 UNAIDS report, the global incidence of HIV infection in 2023 declined by 39% compared with 2010. Nonetheless, approximately 1.3 million [1.0–1.7 million] new HIV infections were reported in 2023 – over three times the 2025 global target of fewer than 370 000 new infections. As the HIV epidemic enters its fifth decade, substantial and urgent advances are required to address remaining challenges.

HIV primarily targets the immune system, particularly CD4^+^ T cells, which play a central role in coordinating immune responses. People living with HIV (PLWH) typically experience progressive depletion of CD4^+^ T cells, sustained immune activation, and systemic inflammation.^6^ The progressive weakening of the immune system increases the risk of contracting various opportunistic infections and developing some types of cancer in PLWH.^7^ ART gradually enhances the level of CD4^+^ T cells and improves the immune function of PLWH. However, approximately 15%–30% of patients do not exhibit full immune reconstitution despite prolonged maintenance of viral suppression. These patients are termed immunological non‐responders (INRs).^8^ Although there is no universally accepted definition of poor immune reconstitution, it is commonly defined as a CD4^+^ T cell count that fails to reach 350 cells/µL after at least 3 years of continuous and effective ART.^9^, ^10^ Multiple studies have sought to elucidate the underlying mechanisms of incomplete immune recovery in INRs, but most remain hypothetical.^11^, ^12^ Proposed mechanisms include the persistence of viral reservoirs, structural damage to lymphoid tissues, and direct HIV‐mediated destruction of CD4^+^ T cells.^13^ Therefore, it is crucial to establish simple and reliable methods to distinguish immunological responders (IRs) from INRs, clarify the mechanisms of immune failure, and identify potential therapeutic strategies.

Several genes involved in the immune response to HIV have been identified.^4^, ^14^, ^15^ However, pinpointing specific target genes remains challenging due to the complexity and heterogeneity of the virus. The interaction between HIV and the host immune system is multifaceted, involving numerous cell types and intricate molecular pathways. Genetic variations in certain genes have been shown to influence host immune responses. For instance, human leukocyte antigen (HLA) genes, which encode proteins responsible for antigen presentation, are strongly associated with susceptibility to HIV infection. Different HLA genotypes can modulate immune responses in PLWH. Soria et al.^16^ identified differential HLA genotypes between IRs and INRs, reporting that a reduced frequency of the inhibitory KIR2DL3 genotype in INRs may impair natural killer (NK) cell function, promote immune activation, and hinder the clearance of latently infected cells, thereby obstructing immune recovery during ART. Similarly, CCR5 and CXCR4, which encode essential co‐receptors for HIV entry into host cells, are key determinants of immune reconstitution. The CCR5 Δ32 mutation, which disrupts co‐receptor function, has been shown to significantly influence immune recovery in PLWH undergoing ART.^17^, ^18^, ^19^ Another study revealed that IL18 G variant alleles and genotypes may promote immune recovery in HIV‐infected individuals receiving ART.^20^ Furthermore, the interferon (IFN) gene family encodes a class of immune‐regulatory proteins involved in resisting viral infections and modulating immune responses. Previous studies have examined the therapeutic effects of various types of IFNs, such as IFN‐α and IFN‐β on HIV.^21^, ^22^ Analysing these genes and gene families could enhance our under‐standing of the immune response mechanisms in PLWH, offering potential targets for the development of HIV vaccines and therapies.

This study aims to investigate the underlying mechanisms contributing to poor immune recovery following ART in PLWH by performing multi‐layered bioinformatics analyses using publicly available transcriptomic datasets from the Gene Expression Omnibus (GEO).In parallel, we conducted flow cytometry–based immunophenotyping on peripheral blood samples from an independent cohort comprising IRs, INRs, and treatment‐naïve patients (TNPs), to characterise functional subsets of T and B lymphocytes. By integrating insights from both computational and experimental approaches, this study seeks to delineate the immunological features associated with divergent responses to ART and to identify potential biomarkers and therapeutic targets to enhance immune reconstitution in PLWH.

## 2 MATERIALS AND METHODS

### 2.1 Data processing and identification of differentially expressed genes (DEGs)

The gene expression datasets of IRs and INRs among PLWH were retrieved from the GEO database (https://www.ncbi.nlm.nih.gov/geo/). The GSE143742 dataset (17 IR samples and 44 INR samples) and the GSE106792 dataset (12 IR samples and 12 INR samples) were derived from the GPL10558 Illumina HumanHT‐12 V4.0 expression bead chip.^23^ IRs were defined as the CD4^+^ T cell counts above 500 cells/µL and INRs were defined as the CD4^+^ T cell counts below 350 cells/µL after receiving ART for at least 2 or 3 years. The detailed clinical information of two datasets was provided in Table S1. The expression data of 1660 metabolism‐related genes were obtained from a published article (PMID: 33181091), the authors integrated multi‐omics data (*n* = 465, the largest dataset to date) to identify 1660 human metabolic genes from 86 metabolic pathways.^24^

The GSE143742 and GSE106792 datasets were merged. The batch effects were removed using the R packages ‘limma’ and ‘sva’. This decreases the differences between datasets caused by experimental errors, allowing multiple datasets to be recombined such that downstream analysis can examine only biological differences. The ‘preprocessCore’ R package was used to normalise the dataset and consequently eliminate the adverse effects caused by outlier sample data. The DEGs were filtered using the ‘limma’ R package based on the following criteria: adj.*p* < .05 and |log2 fold‐change (FC)| > .25. The results of DEGs analysis were displayed using volcano plots and heatmaps.

### 2.2 Functional enrichment analysis

To further examine the functional and biological significance of DEGs in PLWH, DEGs were subjected to GO annotation and KEGG enrichment analysis using the R package ‘clusterprofiler’. The results were ranked based on GeneRatio values within each pathway and visualised using circle plots, where circle colour represents the corresponding *p*‐value.

### 2.3 Establishment of 100 prediction models and hub gene screening

This study constructed 100 predictive models using GSE143742 as the training set and GSE106792 and the merged dataset as the validation set. Furthermore, the concordance index (C‐index) was calculated for each model across all validation datasets to evaluate their predictive capabilities. ROC curves were generated for five hub genes based on the training and validation sets. The area under the curve (AUC) was determined to assess the ability of different genes to predict disease occurrence using the R package ‘pROC’.

### 2.4 Assessment of immune cell infiltration

Immune cell infiltration levels were assessed using the gene set variation analysis (GSVA) R package with the single‐sample gene set enrichment analysis (ssGSEA) function. ssGSEA defines the immune infiltration status between the IR and INR sample groups by calculating normalised enrichment scores. The ‘ggplot2’ package (https://sourceforge.net/projects/ggplot2.mirror/) was used to analyse the results. Differences were considered significant at *p* < .05.

### 2.5 Gene set enrichment analysis

GSEA has been widely utilised to identify potential signalling pathways.^25^, ^26^ To further examine the correlation between the five hub genes and other genes, GSEA software was used to perform GSEA. The top 20 results of single‐gene GSEA analysis were displayed in the Reactome pathway. Differences were considered significant at adjusted *p* < .05.

### 2.6 Clustering analysis

Unsupervised clustering based on the expression of the five hub genes was performed using the R package ‘ConsensusClusterPlus’ to identify metabolism‐related molecular subtypes.^27^ Chi‐squared tests were used to assess associations between clusters and categorical variables. A heatmap was generated using the ‘pheatmap’ R package to visualise correlations among clinical features, gene expression levels, and identified subtypes. KEGG and Reactome pathway gene sets were downloaded from the MSigDB database and scored using the R package GSVA.^28^, ^29^

### 2.7 Quantification real‐time polymerase chain reaction (qRT‐PCR)

The HIV‐infected patients receiving ART with virologic control (HIV‐RNA < 20 copies/mL) for more than 7 years, including 8 HIV‐IRs (CD4^+^ T cells counts above 500 cells/µL) and 8 HIV‐INRs (CD4^+^ T cells counts below 350 cells/µL) were recruited. This study was reviewed and approved by the institutional review board of the National Center for AIDS/STD Control and Prevention, China CDC. Additionally, all study participants provided written informed consent at the time of sample collection.

Total RNA was extracted from 5 mL whole blood using the RNeasy Mini Kit (QIAGEN, USA). Besides, the PrimeScript RT reagent Kit (Takara, Japan) was used to reverse RNA to cDNA and TB Green Premix (Takara, Japan) was utilised to amplify DNA. The sequence of primers is listed in Table S2. The following thermocycling conditions were used for qPCR: 40 cycles, each lasting 5 s at 95°C for denaturation, 30 s at 60°C for annealing and extension. The relative mRNA level of five DEGs was normalised to the endogenous control GAPDH by applying the 2−ΔΔCq method. Statistical comparisons between the two groups were performed using appropriate tests, with *p* < .05 considered statistically significant.

### 2.8 Measurement of immune response by flow cytometry

A total of 71 cryopreserved peripheral blood mononuclear cell (PBMC) samples were analysed by flow cytometry, including 25 HIV‐IRs, 24 HIV‐INRs, 13 treatment‐naïve patients (TNPs), and 9 healthy controls (HCs). PBMCs were thawed and washed with phosphate‐buffered saline (PBS), then resuspended in a calculated volume of PBS (100 µL minus the combined volume of antibody and buffer) per 1 × 10^6^ cells. A surface antibody cocktail was prepared in microcentrifuge tubes by mixing Brilliant Stain Buffer Plus (BD Biosciences) with optimised volumes of fluorochrome‐conjugated antibodies targeting surface markers including anti‐BUV805‐CD3, anti‐AF700‐CD4, anti‐BUV496‐CD8, anti‐BB630‐CD19, anti‐BV750‐CD27, anti‐BUV661‐CXCR5, anti‐PE‐Cy7‐CCR6, anti‐PE‐CF594‐PD‐1, anti‐BUV563‐CD38, anti‐BV711‐HLA‐DR, anti‐PE‐CCR4, anti‐BV605‐CD28, anti‐BV480‐CXCR3 (all from BD Biosciences). A surface antibody cocktail was prepared by mixing Brilliant Stain Buffer Plus (BD Biosciences) with optimised amounts of fluorochrome‐conjugated antibodies targeting surface markers, including anti‐BUV805‐CD3, anti‐AF700‐CD4, anti‐BUV496‐CD8, anti‐BB630‐CD19, anti‐BV750‐CD27, anti‐BUV661‐CXCR5, anti‐PE‐Cy7‐CCR6, anti‐PE‐CF594‐PD‐1, anti‐BUV563‐CD38, anti‐BV711‐HLA‐DR, anti‐PE‐CCR4, anti‐BV605‐CD28, and anti‐BV480‐CXCR3 (all from BD Biosciences). The antibody cocktail was added to each sample and incubated for 30 min at room temperature in the dark. Cells were then washed twice with PBS (130 µL and 230 µL, respectively; centrifuged at 400 × *g* for 5 min) and incubated with LIVE/DEAD™ Fixable Viability Dye (Invitrogen) for 15 min at room temperature in the dark. Two additional PBS washes were performed to remove excess dye. Finally, cells were resuspended in PBS and acquired using a FACSymphony™ A5 flow cytometer (BD Biosciences). Data were analysed using FlowJo software (version 10.8.1).

### 2.9 Statistical analyses

Data and statistical analyses were performed using R v4.2.2 and Prism v9.5.0. Categorical variables were compared using the *t*‐test and two‐tailed non‐parametric Mann–Whitney U test. *p* < .05 indicated statistical significance.

## 3 RESULTS

### 3.1 Screening and functional analysis of DEGs between IRs and INRs

**FIGURE 1:**
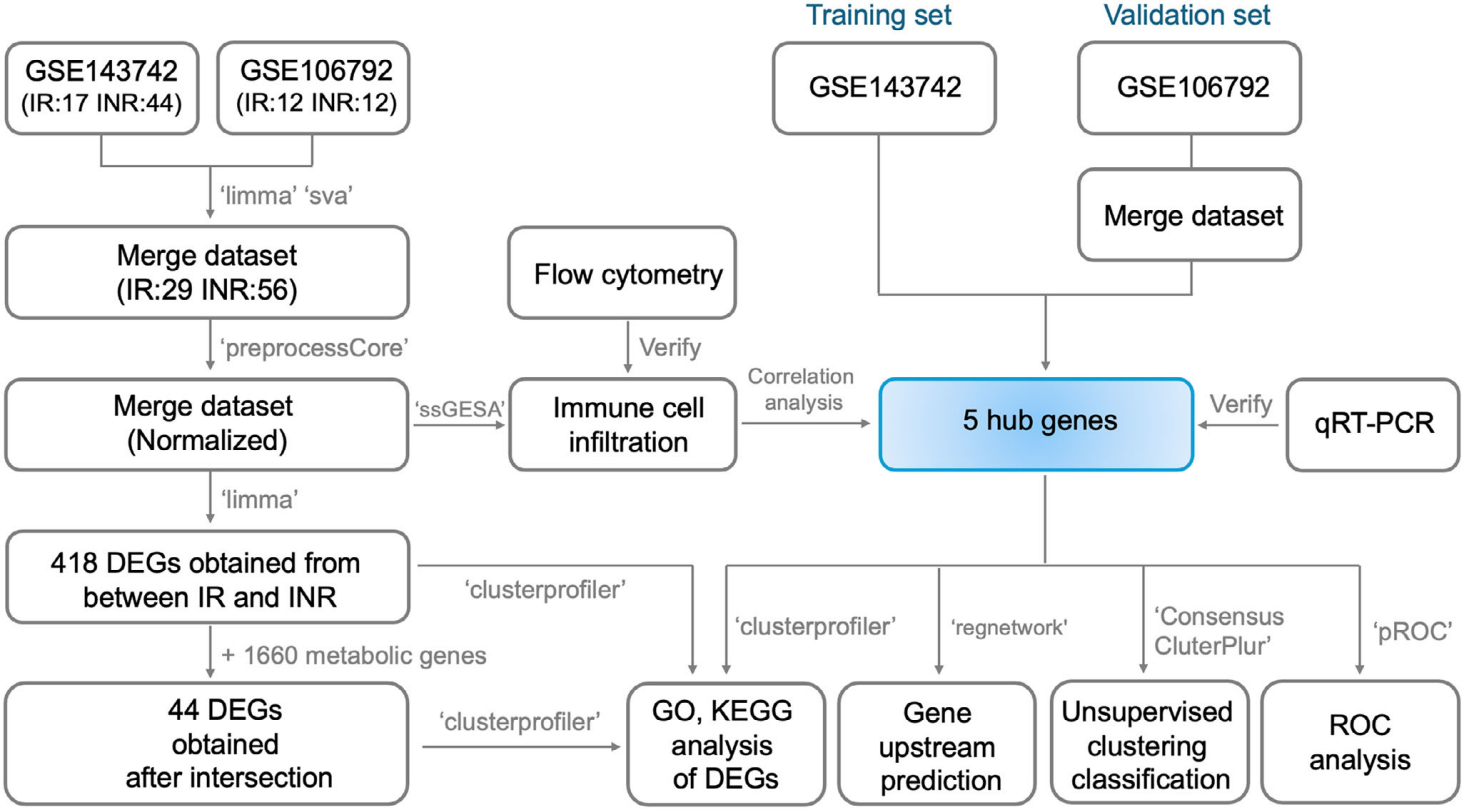
Schematic diagram of the study design.

**FIGURE 2:**
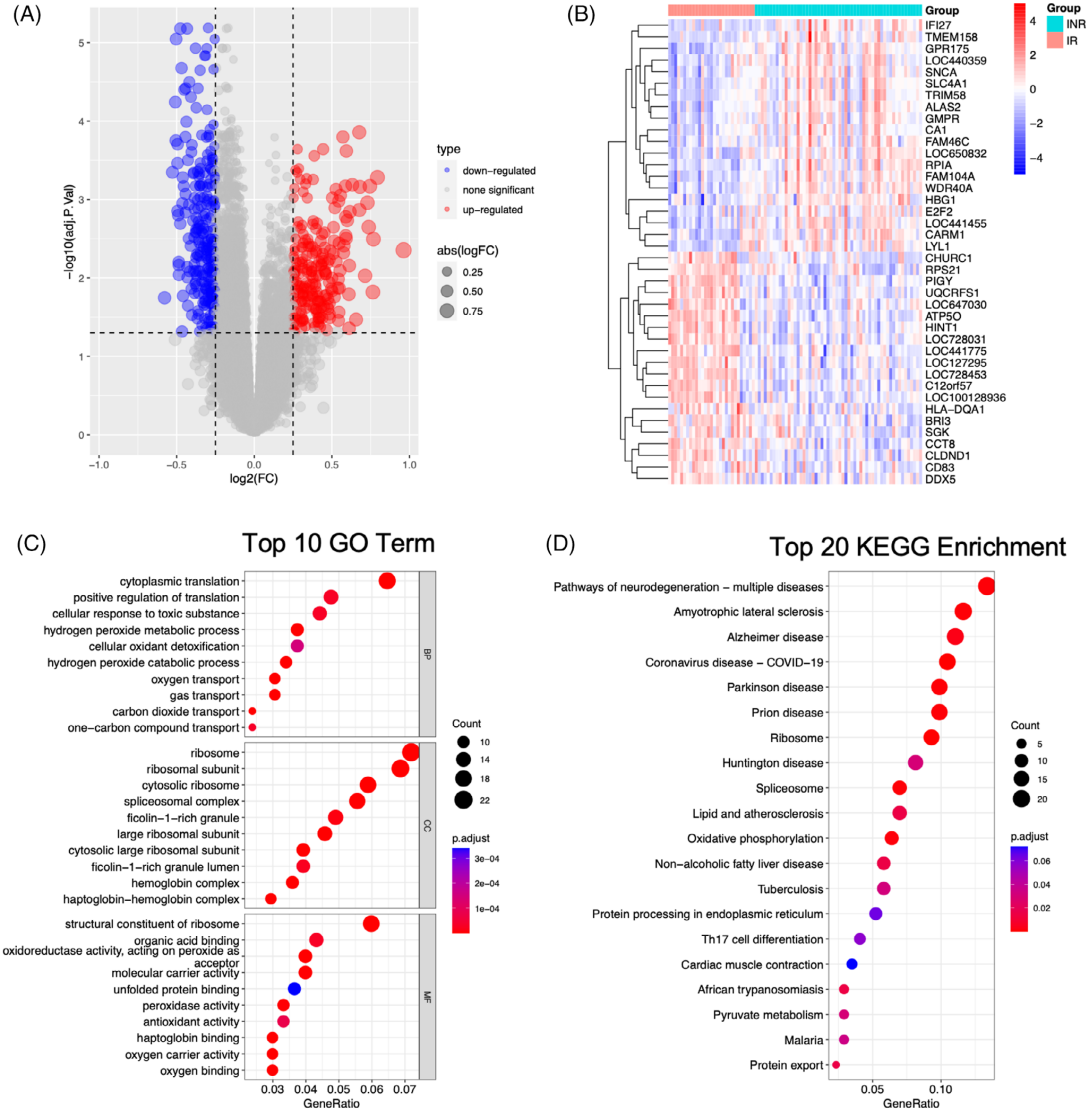
Differential gene expression and pathway enrichment analysis. (A) Volcano plot of DEGs (blue and red represent downregulated and upregulated genes, respectively). (B) Heatmap illustrating the Top 20 DEGs between IR and INR groups (red and blue indicate upregulation and downregulation, respectively). (C) GO analysis results show the genes annotated in biological process (BP), cellular component (CC), and molecular function (MF). (D) Results of KEGG pathway enrichment analysis. All pathways were sorted according to GeneRatio with the size of the circles representing the number of enriched genes and the colour indicating the *p*.adjust value.

Figure 1 illustrates the overall research workflow. Following the integration of the GSE143742 and GSE106792 datasets, gene expression data for 31 319 genes across 85 samples (29 IRs and 56 INRs) were obtained. After normalisation, differential expression analysis identified 219 upregulated and 259 downregulated genes (Figure S1 and Table S3). The volcano plot and heatmap of the DEGs are presented in Figure 2A and B. The gene expression profiles were significantly different between the IR and INR groups. To investigate the biological roles of these DEGs, functional enrichment analyses were conducted. GO analysis revealed the enrichment of DEGs in molecular function (MF), cellular component (CC), and biological process (BP). DEGs between the IR and INR groups were enriched in the following GO terms: MF term, structural constituent of ribosome, molecular adaptor activity, and organic acid‐binding; CC term, ribosome, ribosomal subunit, and mitochondrial inner membrane. Mitochondria are the cellular metabolic centre, while ribosomes are sites of protein synthesis.^30^, ^31^ The enrichment of DEGs in the mitochondria and ribosomes prompted the evaluation of the metabolic processes in the IR and INR groups (Figure 2C). Additionally, KEGG enrichment analysis revealed that the DEGs were enriched in signalling pathways related to neurodegenerative diseases, amyotrophic lateral sclerosis, Alzheimer's disease, COVID‐19, measles, and Parkinson's disease (Figure 2D). In addition to providing gene sets, KEGG analysis enables the elucidation of complex interactions between genes and metabolites.

### 3.2 Identification of metabolism‐related genes

**FIGURE 3:**
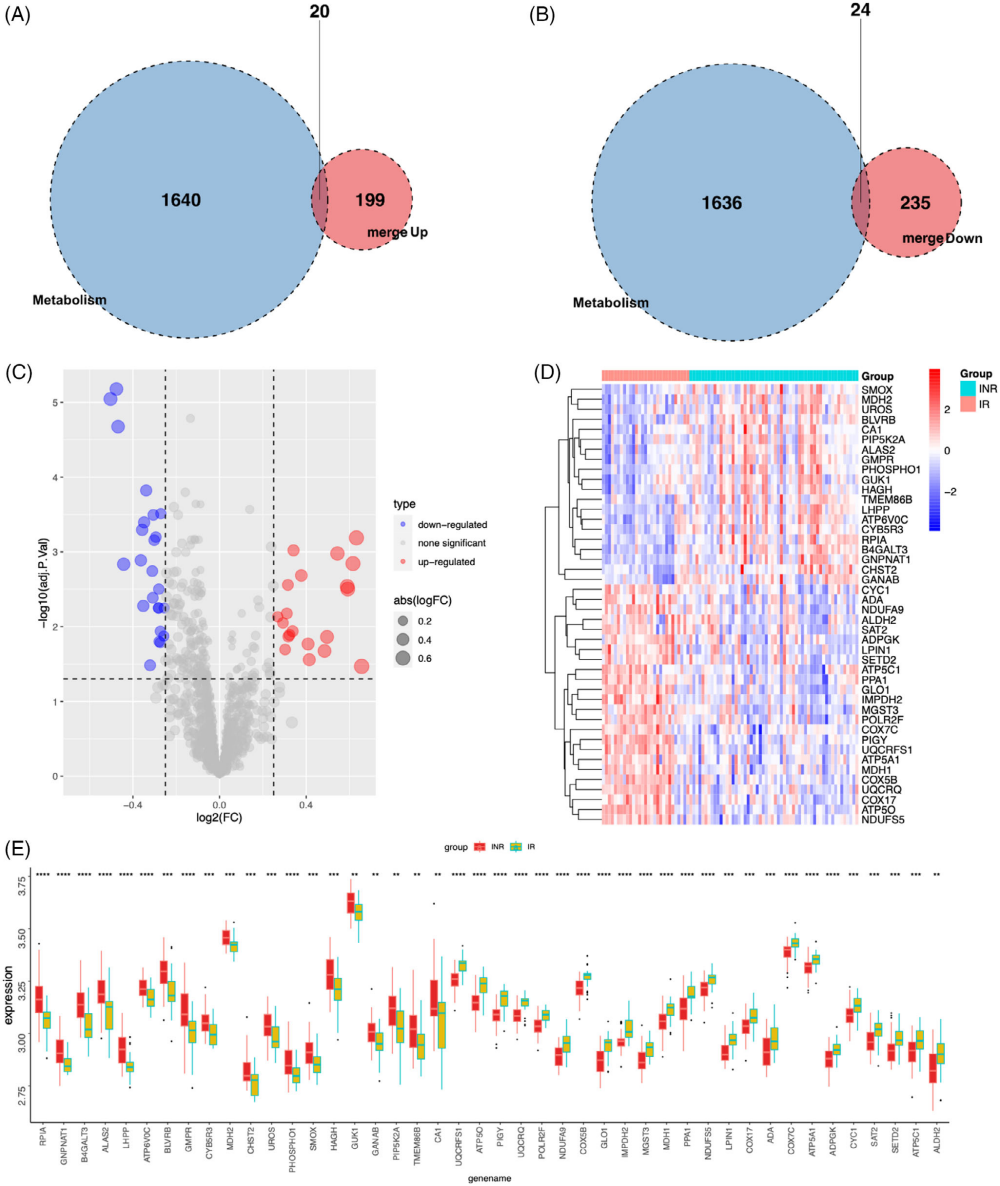
(A, B) Intersection of the differentially expressed genes with the metabolic gene set. The intersection of the upregulated differentially expressed genes with the metabolic gene set, yielding 20 DEGs, is shown in A. The intersection of the downregulated differentially expressed genes with the metabolic gene set, yielding 24 DEGs, is shown in B. (C, D) The differential expression patterns of 44 DEGs obtained after intersecting the initial DEGs with the metabolic genes between IR and INR groups. DEGs are presented separately using a volcano plot (C) and a heatmap (D). (E) The expression patterns of 44 DEGs between IR and INR groups are illustrated using box plots (plotted using the ‘ggplot2’ package). *p < .05; **p < .01; and ***p < .001; ****p < .0001; ns not significant.

Cellular metabolism plays a critical role in determining the fate and functional activity of immune cells. In the context of HIV infection, understanding the metabolic underpinnings of immune responses is essential. Clerc et al.^32^ demonstrated that HIV‐1 infection alters the glucose metabolism of immune cells. In this study, the previously identified 219 upregulated and 259 downregulated DEGs were intersected with a set of 1660 metabolism‐related genes, resulting in the identification of 44 metabolism‐associated DEGs (Figure 3A and B). The volcano plot and heatmap revealed the differential expression of these 44 DEGs between the IR and INR groups (Figure 3C and D). Next, box plots were used to visually represent the differential expression of these 44 metabolism‐related DEGs between the IR and INR groups (Figure 3E). Among these genes, the expression of 20 genes, such as *RPIA* and *CA1* in the INR group was significantly higher than that in the IR group. Meanwhile, the expression of genes, such as *UQCRFS1* in the IR group was significantly higher than that in the INR group. These 44 DEGs were further subjected to GO and KEGG functional enrichment analysis (Figure S2).

### 3.3 Establishment of prediction models and identification of five key hub genes

**FIGURE 4:**
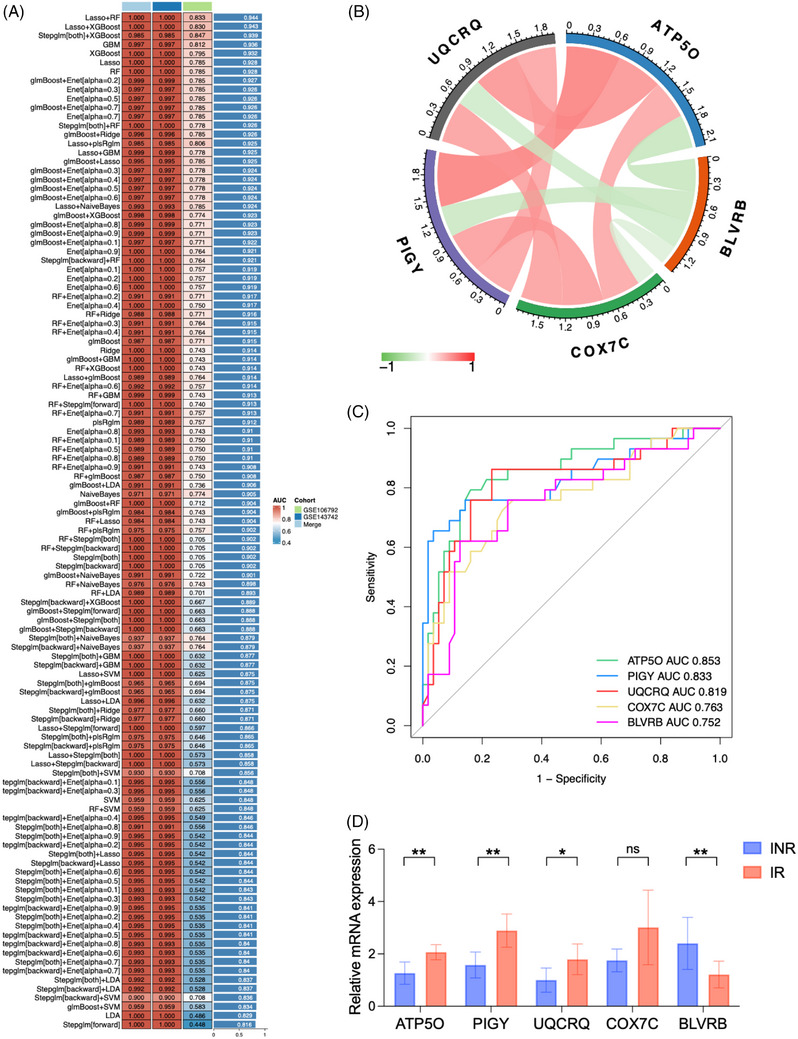
(A) Construction of 100 predictive models. (B) Correlation between the five core genes. Red and green indicate positive and negative correlations, respectively. (C) ROC curves of the five core genes for predicting disease occurrence. The *X*‐axis and *Y*‐axis represent ‘1 – specificity’ and ‘sensitivity’, respectively. (D) The expression levels of five hub genes between IR and INR groups using qRT‐PCR. *p < .05; **p < .01; and ***p < .001; ****p < .0001; ns not significant.

Using the GSE143742 dataset as the training set and both the GSE106792 and merged datasets as validation sets, 100 predictive models were constructed. The concordance index (C‐index) was calculated for each model to assess predictive performance across validation datasets. Among these, the LASSO combined with Random Forest (LASSO + RF) model demonstrated the highest average C‐index (Figure 4A). Based on LASSO + RF selection, five genes (*PIGY, UQCRQ, ATP5O, BLVRB*, and *COX7C*) were identified as key hub genes in this study. The correlation between these five genes was analysed (Figure 4B). The expression of *BLVRB* was negatively correlated with that of the other four genes. R language was used to plot ROC curves for predicting disease occurrence using these five genes, and the AUC value was calculated for each gene. *ATP5O* exhibited the highest AUC value (0.853), followed by *PIGY* (AUC = 0.833), *UQCRQ* (AUC = 0.819), *COX7C* (AUC = 0.763), and *BLVRB* (AUC = 0.752) (Figure 4C). In vitro qRT‐PCR experiments confirmed that there were statistical differences in the expression levels of *ATP50, PIGY, UQCRQ*, and *BLVRB* between the IR and INR groups (Figure 4D). Further exploration of the biological functions of these genes is of great significance for understanding the diagnosis and treatment of PLWH.

### 3.4 Infiltrating immune cell analysis

**FIGURE 5:**
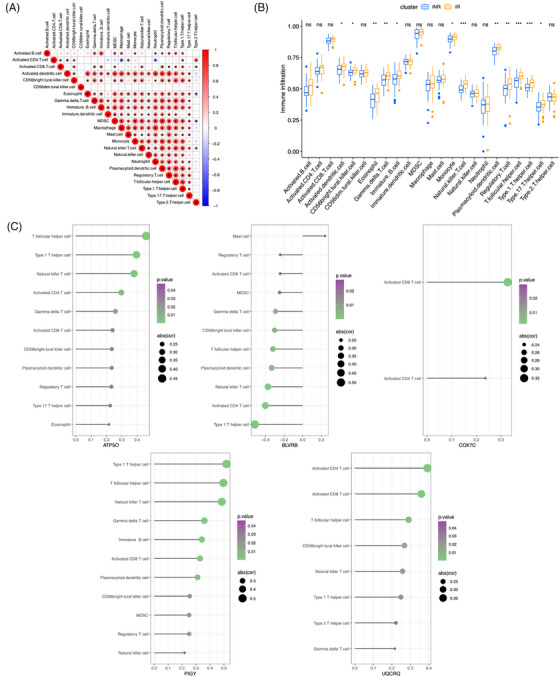
(A) Correlation of the proportions of immune cell infiltration. (B) Differential immune cell infiltration levels between the immunological responder (IR) and immunological non‐responder (INR) groups. *p < .05; **p < .01; and ***p < .001; ****p < .0001; ns not significant. (C) Correlation between the five core genes and immune cell infiltration (only displaying immune cells with *p* < .05). The size of the circles represents the magnitude of the correlation coefficient, while the colour represents the *p*‐value.

The infiltration levels of 23 different types of immune cells varied between the IR and INR groups. Correlation analysis among these immune cell types revealed that the proportion of activated dendritic cells (DCs) was positively correlated with that of eosinophils, myeloid‐derived suppressor cells, macrophages, monocytes, neutrophils, plasmacytoid DCs, regulatory T cells, and several other subsets (Figure 5A). In contrast, the proportion of activated CD4⁺ T cells was negatively correlated with that of macrophages and neutrophils. Overall, immune cell infiltration showed an upward trend in the IR group relative to the INR group. Notably, 13 immune cell subsets – including natural killer T (NKT) cells, T follicular helper (Tfh) cells, and T helper 1 (Th1) cells – were significantly more abundant in the IR group (Figure 5B).

Next, the correlation between the five core genes and immune cell infiltration was examined (Figure 5C). The expression levels of *ATP5O* and *PIGY* were significantly and positively correlated with the proportion of Tfh cells, Th1 cells, and NKT cells. Meanwhile, the expression levels of *UQCRQ* and *COX7C* were significantly and positively correlated with the proportion of activated CD8^+^ T cells. Additionally, the expression of *UQCRQ* was significantly and positively correlated with the proportion of activated CD4^+^ T cells. In contrast, the expression of *BLVRB* was significantly and negatively correlated with the proportion of Th1 cells, activated CD4^+^ T cells, and NKT cells.

**FIGURE 6:**
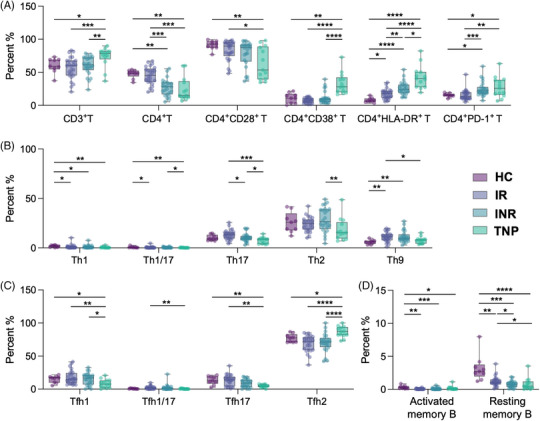
(A) The proportions of CD3⁺ T cells, CD4⁺ T cells, and CD4⁺ T cells expressing activation or exhaustion markers (CD28⁺, CD38⁺, HLA‐DR⁺, and PD‐1⁺) were analysed across healthy controls (HC), immunological responders (IR), immunological non‐responders (INR), and treatment‐naïve patients (TNP). (B) The distribution of T helper (Th) cell subsets was compared among the four groups. (C) The proportions of T follicular helper (Tfh) cell subsets were assessed in HC, IR, INR, and TNP individuals. (D) The frequencies of resting and activated B cells were evaluated across the same groups. Multiple group differences were analysed using the two‐tailed non‐parametric Kruskal–Wallis test. ****p < .0001, *** *p* < .001, ** *p* < .01, * *p* < .05, ns not significant.

This study also systematically evaluated T and B cell functional subsets across HIV‐infected individuals with different immune outcomes, including IR, INR, and treatment‐naïve patient (TNP) (Figures 6 and S3). CD3⁺ T cell levels were similar between IR and INR but elevated in TNP. CD4⁺ T cells were significantly lower in INR and TNP than in IR and healthy controls (HC); TNP also showed decreased CD4⁺ CD28⁺ and increased CD4⁺ CD38⁺ T cells. CD4⁺ HLA‐DR⁺ T cells progressively increased from IR to INR and TNP, while CD4⁺ PD‐1⁺ T cells were significantly upregulated in INR and TNP, indicating chronic activation and exhaustion (Figure 6A). Tfh expression in IR resembled HC, but Th1, Th17, and Th9 subsets differed. INR showed marked reductions in Tfh17, Th1, Th17, and Th9 subsets, and TNP exhibited even more profound declines in Tfh1, Th1/17, Th17, and Th2 subsets, suggesting broad immune dysfunction (Figure 6B and C). Resting memory B cells were significantly reduced in INR compared to IR and HC, while activated memory B cells were comparable (Figure 6D). These findings indicate impaired long‐term immune memory in INR individuals and incomplete B cell recovery even in IR, underscoring the complexity of immune reconstitution after antiretroviral therapy.

### 3.5 Single‐gene GSEA analyses

The correlation between *PIGY, UQCRQ, ATP5O, BLVRB*, and *COX7C* and the top 50 positively and negatively correlated genes was examined (Figures S4 and S5). GSEA revealed the signalling pathways in which these five key genes were enriched. The top 20 results are shown in Figure S6. Among the five genes, only BLVRB exhibited significant negative enrichment in pathways such as the late phase of the HIV life cycle, cellular response to heat stress, and the overall HIV life cycle. These findings suggest that BLVRB may play an inhibitory role in the immune reconstitution process in PLWH.

### 3.6 Molecular stratification reveals transcriptionally distinct subtypes driven by metabolism‐associated hub genes

**FIGURE 7:**
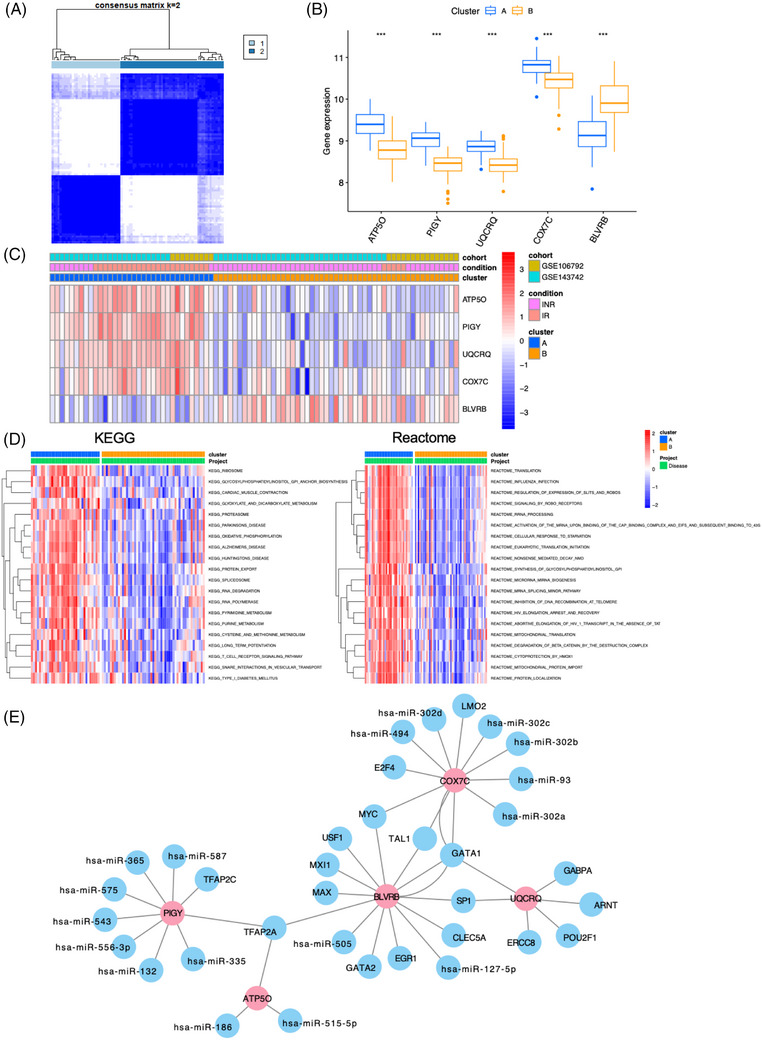
(A) Unsupervised clustering based on the five core genes using the R package ‘X’. The optimal number of clusters was two (*k* = 2). (B) Differential expression of the five core genes between different subtypes. ****p < .0001, *** *p* < .001, ** *p* < .01, * *p* < .05, ns not significant. (C) The R package ‘pheatmap’ was used to generate a heatmap to illustrate the correlation between clinical features, gene expression, and subtypes. (D) KEGG and Reactome pathways were downloaded from the Msigdb database and scored using the R package ‘GSVA’. The R package ‘pheatmap’ was used to generate comparative heatmaps between the two groups. (E) The microRNAs (miRNAs) and transcription factors upstream of the genes. Red indicates core genes.

To delineate transcriptional subtypes associated with metabolic remodelling, unsupervised consensus clustering was performed based on the expression profiles of the five metabolism‐associated hub genes (*ATP5O, PIGY, UQCRQ, COX7C, BLVRB*). The analysis stratified the integrated dataset into two robust molecular subtypes (Cluster A, *n* = 34; Cluster B, *n* = 51), with optimal separation achieved at *k* = 2 (Figure 7A). Principal component analysis (PCA) demonstrated clear segregation between clusters, confirming subtype‐specific transcriptional architecture (Figure S7A). Subtype‐specific expression profiling revealed that *ATP5O, PIGY, UQCRQ*, and *COX7C* were markedly upregulated in Cluster A, whereas *BLVRB* was predominantly enriched in Cluster B (Figure 7B). A composite heatmap integrating cohort origin, clinical classification (IR vs. INR), and gene expression patterns further confirmed the coherence between molecular subtype and immune phenotype (Figure 7C). These findings suggest that metabolic gene expression signatures underlie distinct immunometabolism states in PLWH. To evaluate the functional divergence between subtypes, pathway activity scores were computed using curated KEGG and Reactome gene sets. Cluster A exhibited broad upregulation of immune activation and mitochondrial energy metabolism pathways, while Cluster B was characterised by transcriptional repression of these modules (Figure 7D). Differential gene expression analysis between clusters identified a subset of cluster‐enriched transcripts, which were further subjected to GO and KEGG enrichment using clusterProfiler. These cluster‐specific DEGs were significantly associated with oxidative phosphorylation, ribosomal activity, and T cell activation (Figure S7B–D), reinforcing the immunometabolism distinction between the two subtypes.

To elucidate upstream regulatory circuits shaping these transcriptional states, regulatory network inference was performed using the RegNetwork database. Cytoscape‐based visualisation revealed convergence of several transcription factors (TFs) on multiple hub genes (Figure 7E). TFAP2A was identified as a shared upstream regulator of *PIGY, BLVRB*, and *ATP5O*, while GATA1 regulated *BLVRB, UQCRQ*, and *COX7C*, suggesting coordinated transcriptional control across metabolic and immunological axes. These results support the presence of discrete metabolic programs in PLWH, orchestrated by shared regulatory networks that may influence immunological responsiveness under ART.

## 4 DISCUSSION

ART has revolutionised the treatment of HIV, significantly enhancing the prognosis and quality of life for PLWH.^33^ However, the response to ART can vary considerably among patients, particularly between IRs and INRs. In fact, continuous ART fails to substantially increase CD4^+^ T cell levels in INRs, leaving them in a state of immune non‐response. This condition heightens their susceptibility to opportunistic infections and contributes to poorer overall outcomes.^34^ Given these challenges, the identification of predictive and diagnostic markers is vital for the early detection of INRs. Such markers may inform the development of clinical strategies aimed at improving treatment outcomes for PLWH, ultimately enhancing their quality of life and health.

The present study sought to elucidate the molecular and immunological factors underlying the variable immune reconstitution among PLWH undergoing ART, specifically focusing on INRs. Through integrated transcriptomic and immunophenotypic analyses, our findings underscore pronounced metabolic dysfunction – particularly within mitochondrial and ribosomal pathways – as a critical factor impeding immune reconstitution. Notably, using advanced machine learning techniques, *ATP5O, PIGY, UQCRQ, COX7C*, and *BLVRB* were robustly identified as pivotal biomarkers distinguishing INRs from IRs, reflecting significant roles in metabolic regulation and immune modulation. Mitochondria are essential for immune cell bioenergetics, playing critical roles in cellular metabolism, energy production, signalling, and immune function.^30^ Previous research has consistently highlighted mitochondrial dysfunction as a prominent factor influencing immune recovery, with compromised oxidative phosphorylation (OXPHOS) linked directly to impaired T cell functionality.^23^ In line with these findings, we observed marked downregulation of *ATP5O* and *UQCRQ* – integral components of mitochondrial electron transport complexes – in INRs. *ATP5O*, a subunit of ATP synthase, is essential for ATP synthesis, and its downregulation potentially limits the ATP supply required for robust immune cell proliferation and survival. Similarly, *UQCRQ*, part of complex III in the electron transport chain, is crucial for efficient mitochondrial respiration; its reduction could lead to increased reactive oxygen species (ROS) production and subsequent oxidative stress, impairing immune cell function.^35^ In contrast, our analysis indicated elevated expression of *BLVRB* in INRs, negatively correlating with crucial immune subsets such as Th1 and NKT cells. *BLVRB*, involved in haeme catabolism and antioxidant defence mechanisms, modulates cellular oxidative stress.^34^ Its overexpression in INRs might reflect a compensatory response to heightened oxidative stress due to impaired mitochondrial function, potentially exacerbating chronic inflammation and T cell exhaustion – hallmarks of inadequate immune recovery. These metabolic perturbations provide novel insights into the pathogenic mechanisms underlying persistent immune dysfunction despite sustained virological suppression. Comparative analysis with recent literature further contextualises our findings within the existing body of HIV research. Younes et al. demonstrated specific mitochondrial dysfunction in cycling CD4^+^ T cells from INRs, highlighting metabolic abnormalities as a critical feature of incomplete immune reconstitution.^23^ This aligns closely with our transcriptomic findings regarding *ATP5O* and *UQCRQ*. Additionally, studies by Ding et al. and Lisco et al. identified immune activation profiles in INRs, albeit with divergent outcomes.^14^, ^36^ These discrepancies likely reflect differences in patient demographics, ART duration, and methodological variations in assessing immune subsets. Our comprehensive integration of bioinformatics and detailed immunophenotyping addresses some prior methodological shortcomings, providing robust, reproducible, and biologically interpretable data. Our comprehensive profiling of T and B cell subsets among IR, INR, and TNP individuals reveals that incomplete immune recovery in INR extends beyond CD4⁺ T cell counts to include persistent immune activation (elevated HLA‐DR⁺, PD‐1⁺), loss of functional Th subsets (notably Th1, Th17, and Tfh17), and impaired B cell memory. While IR patients partially restore Tfh and memory B cells, INR individuals display marked deficits in both T and B cell compartments, consistent with chronic immune dysregulation and exhaustion.^37^, ^38^ The observed depletion of Th17 and Th1 cells, essential for mucosal integrity and viral control, aligns with prior findings linking their loss to microbial translocation and systemic inflammation in INRs.^39^ Furthermore, the reduction in resting memory B cells despite ART supports earlier reports of impaired humoral longevity and memory recall capacity in treated HIV infection.^12^, ^40^ These findings underscore the multifaceted nature of immune failure in INR and highlight the need for adjunctive strategies beyond ART to restore both T and B cell functionality. Importantly, our study also delineated two distinct molecular subtypes characterised by differential expression of the metabolism‐associated hub genes. Subtype stratification revealed Cluster A predominantly represented by IRs, characterised by heightened mitochondrial gene expression, active immune responses, and effective memory T and B cell reconstitution. In contrast, Cluster B, predominantly comprising INRs, exhibited metabolic suppression, profound immune exhaustion, and impaired immune reconstitution. These molecularly distinct subtypes reinforce the critical role of metabolic processes in shaping immune recovery outcomes, underscoring the importance of personalised interventions targeting metabolic pathways to enhance therapeutic efficacy.^24^, ^33^

Furthermore, the identification of upstream regulatory mechanisms further expands the biological interpretation of our findings. The identification of upstream regulators, including TFAP2A and GATA1, offers mechanistic insights into the metabolic‐immune crosstalk underlying our findings. TFAP2A may coordinate mitochondrial and metabolic gene expression (e.g., *ATP5O, PIGY*), while GATA1 appears linked to redox and mitochondrial regulation via *BLVRB* and *COX7C*. These hypotheses warrant further validation through functional and metabolomic studies. Given the likely complexity of metabolic‐immune interactions, future work should also account for genetic and environmental variability to improve mechanistic understanding and translational relevance.

Although our study offers a potential new strategy to identify molecular and immunological differences between IRs and INRs in HIV treatment, there are still several limitations that need to be addressed. Our analysis is based on public databases, but the sample size of the study population is limited, and further optimisation is required in future research. Additionally, while the differentially expressed genes identified have been preliminarily validated, they may not act independently but through complex network interactions that affect immune cell function. Although these genes show potential roles related to immune recovery, the current evidence remains observational. To further validate these genes as potential diagnostic markers for PLWH, larger sample sizes and more in‐depth mechanistic studies are needed to confirm their clinical relevance. Furthermore, future research should consider immune response differences across different geographical regions and racial groups to enhance the generalisability of the results.

## 5 CONCLUSION

This study highlights the distinct gene expression profiles and immune cell infiltration patterns between IRs and INRs. We found that the DEGs were significantly enriched in metabolic pathways, particularly mitochondrial‐related processes. By intersecting these DEGs with known metabolic gene sets, we identified 44 novel DEGs. Subsequent machine learning analysis revealed *ATP5O, PIGY, UQCRQ, COX7C*, and *BLVRB* as key metabolic genes potentially involved in immune dysregulation. Complementing the transcriptomic findings, flow cytometry–based immunophenotyping demonstrated that INRs exhibited impaired differentiation of memory T and B cell subsets, heightened T cell exhaustion, and reduced immune cell infiltration compared to IRs. Specifically, INRs had lower frequencies of CD4⁺ T cells and resting memory B cells, along with increased expression of exhaustion markers such as PD‐1 and HLA‐DR on T cells. These immunological features suggest a state of persistent immune dysfunction despite virologic suppression. Future studies will focus on functional validation of these key genes and further dissect the metabolic–immune interface that governs immune recovery in PLWH.

## AUTHOR CONTRIBUTIONS

QFC, KL, and QXH designed the experiments and wrote the paper. LR and SW analysed and interpreted the data. JGW helped in laboratory and sample collection. YL and JSH made the relevant edits to the manuscript. YMS and DL designed the experiments and revised the full text. All authors read and approved the final manuscript.

## ETHICS STATEMENT

This study was reviewed and approved by the institutional review board of the National Center for AIDS/STD Control and Prevention, China CDC. Additionally, all study participants provided written informed consent at the time of sample collection.

## Supporting information



Supporting Information

Supporting Information

Supporting Information

Supporting Information

Supporting Information

Supporting Information

Supporting Information

Supporting Information

Supporting Information

Supporting Information

## Data Availability

The public datasets were downloaded and analysed in this study, which can be found in GEO data repository and included the accession numbers as follows: GSE143742 and GSE106792. The metabolic gene used in the analysis was derived from the article PMID: 33181091.
