# Supplementary figures and images for: Immune cell subset profiling and metabolic dysregulation define the divergent immune microenvironments in HIV immunological non‐responders
Source: Clin Transl Med. 2025 Oct 13;15(10):e70498. doi: 10.1002/ctm2.70498 (PMC12518780; doi:10.1002/ctm2.70498)

**A**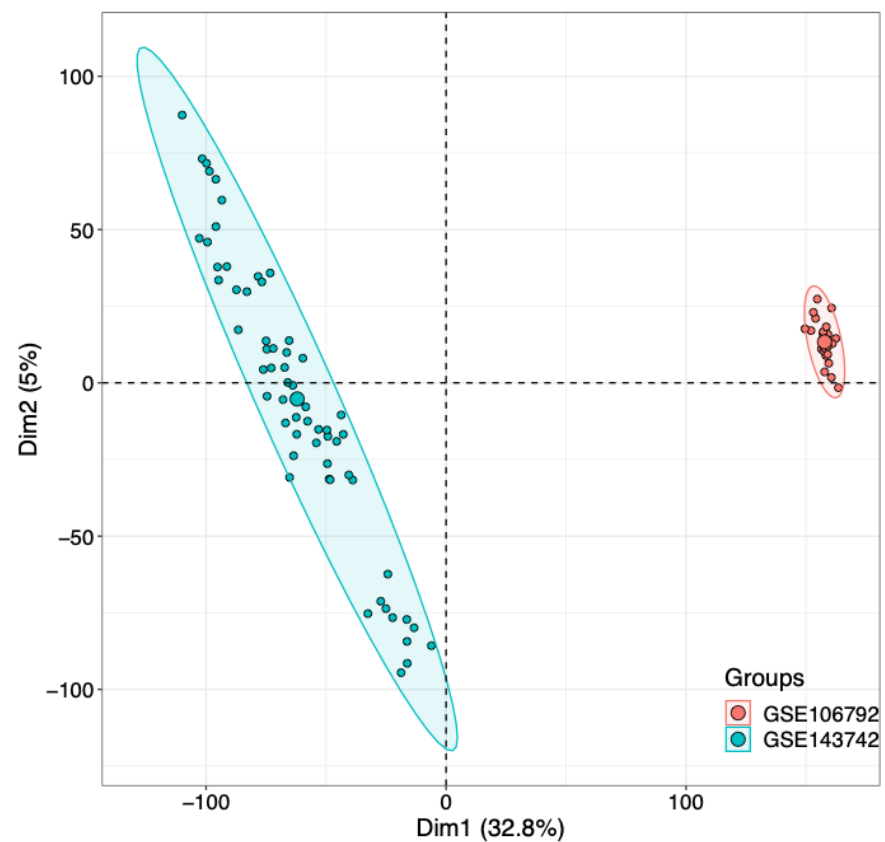**B**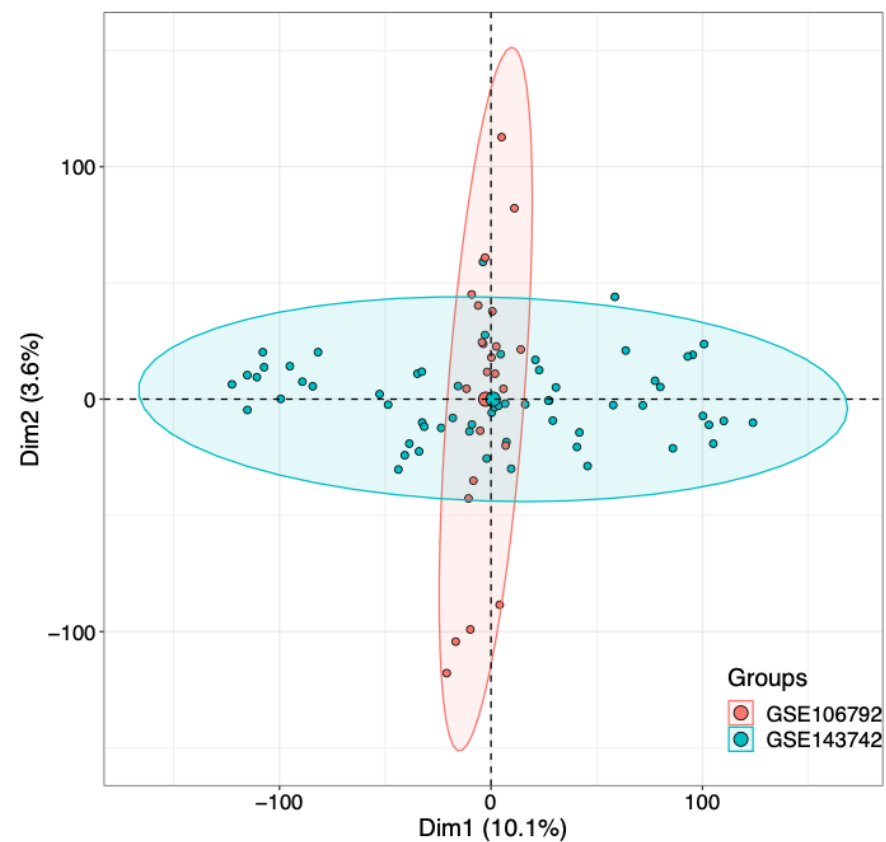**C**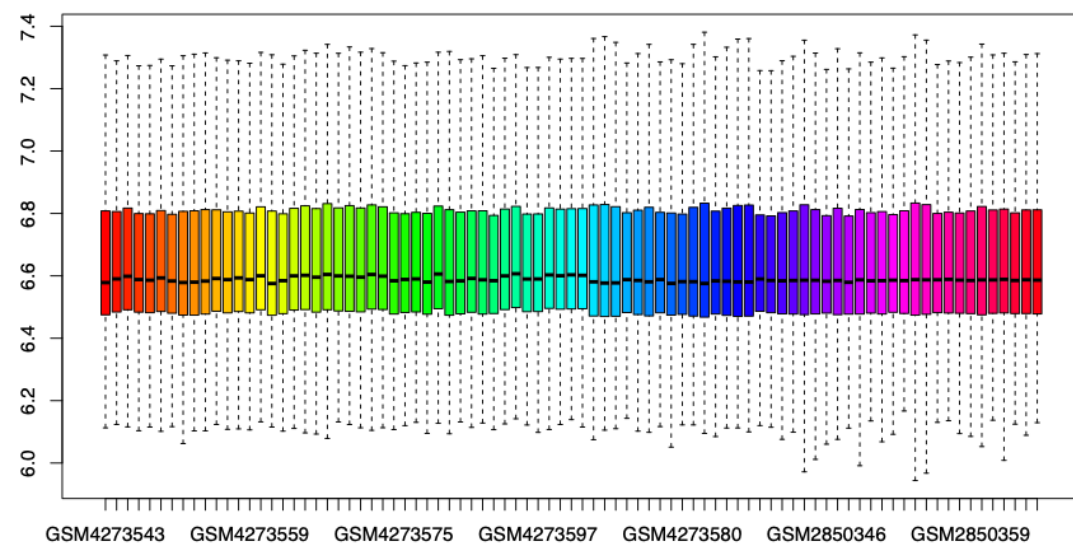**D**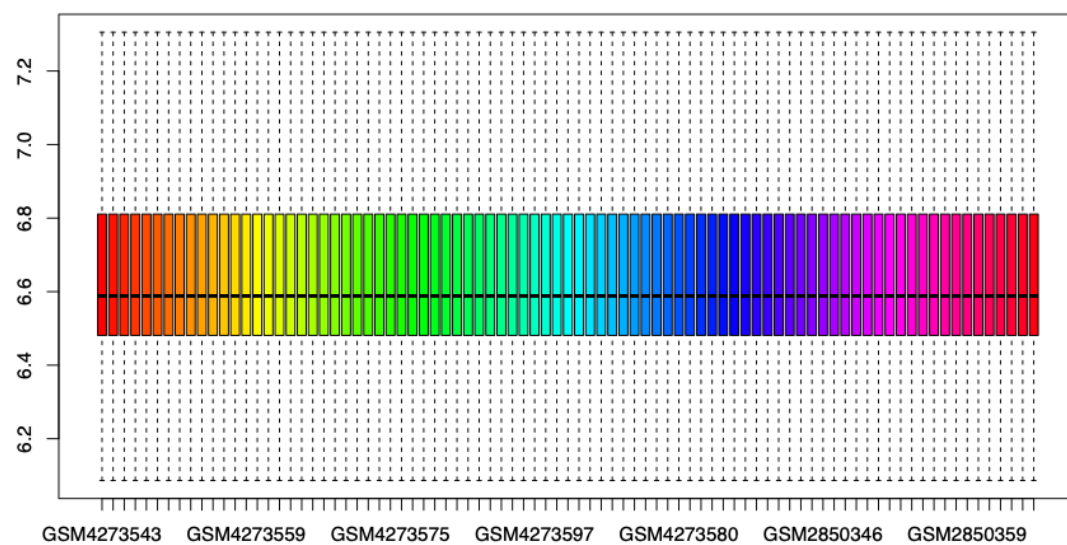

Supplement: Supplementary file 1 — Supporting Information [file CTM2-15-e70498-s001.pdf]

**A** Top 20 GO BP Term

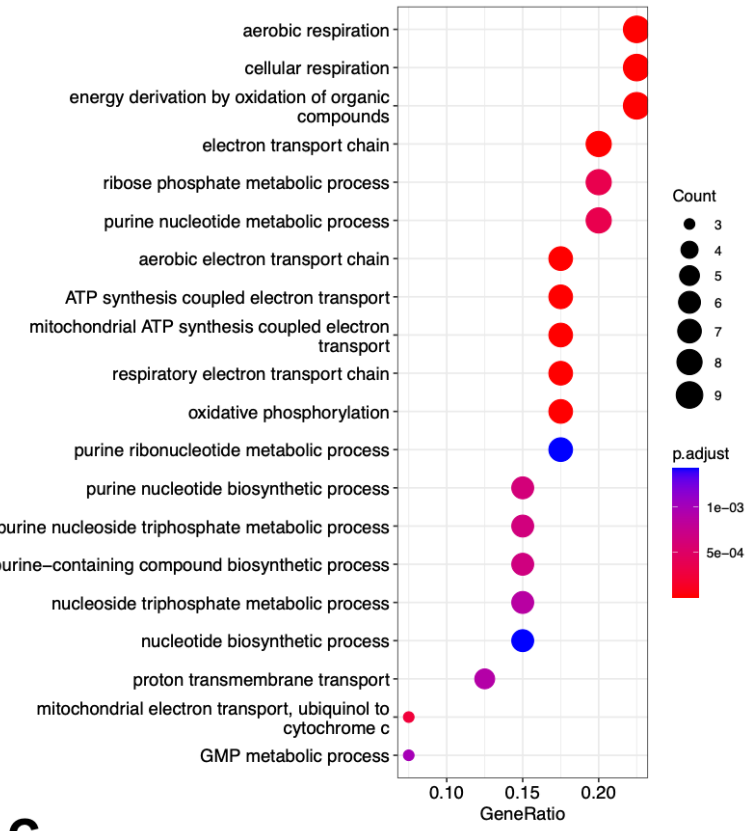

**B** Top 20 GO CC Term

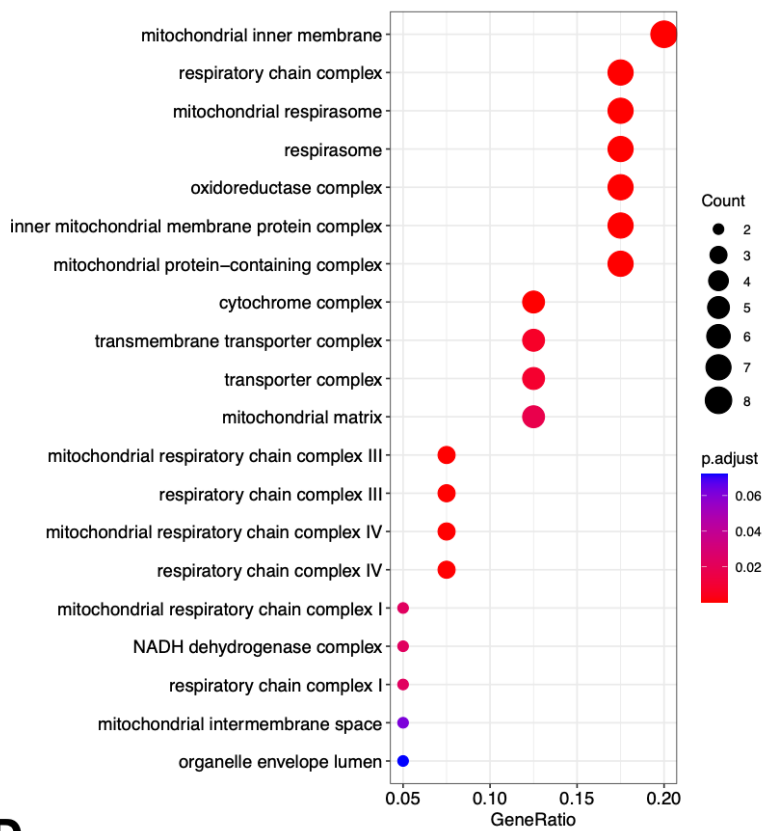

**C** Top 20 GO MF Term

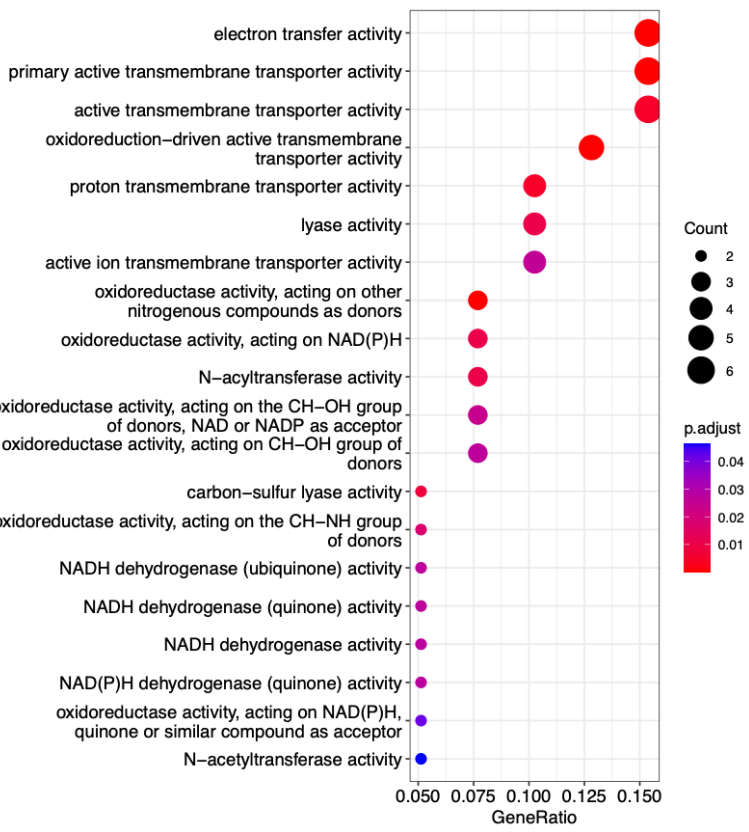

**D** Top 20 KEGG Enrichment

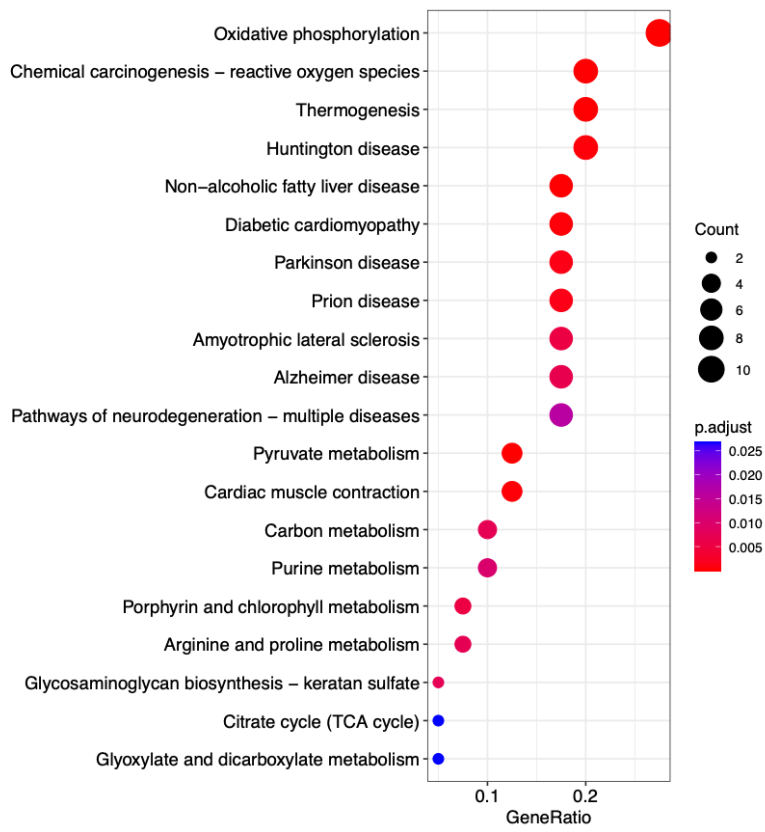

Supplement: Supplementary file 2 — Supporting Information [file CTM2-15-e70498-s005.pdf]

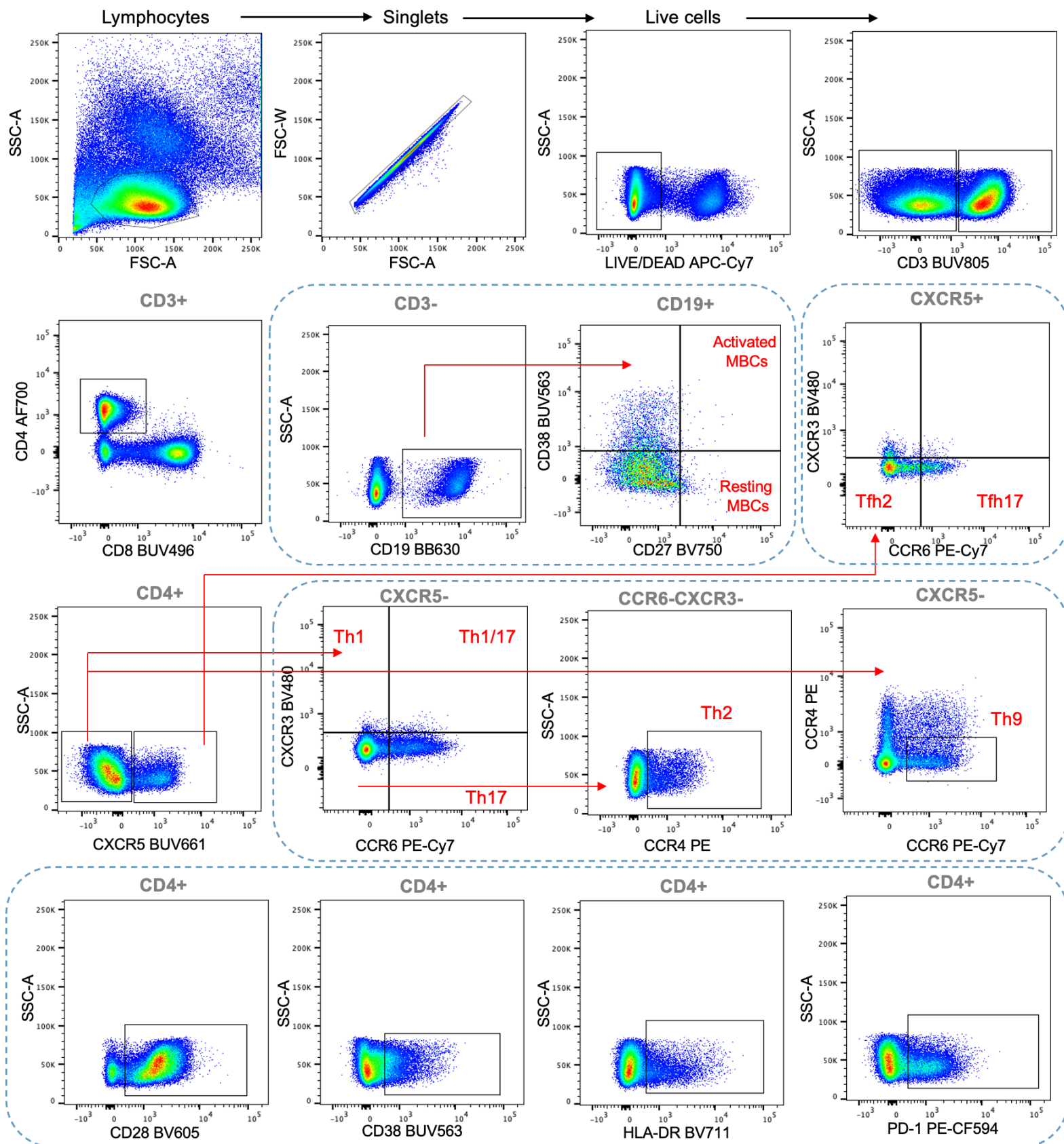

Supplement: Supplementary file 3 — Supporting Information [file CTM2-15-e70498-s010.pdf]

A

ATP5O

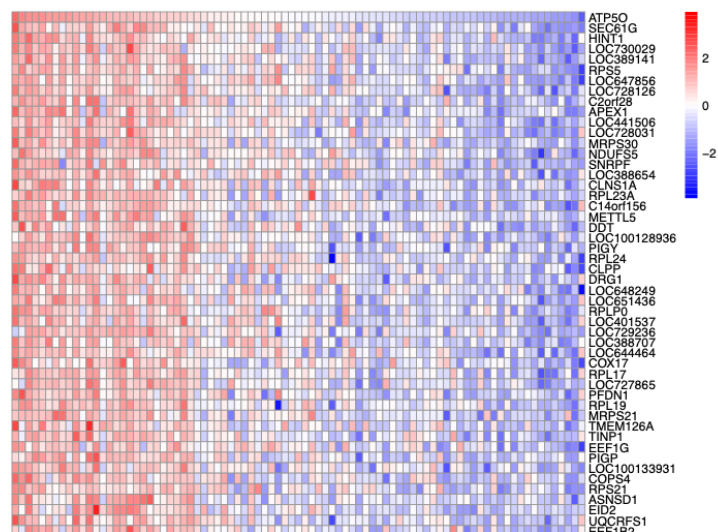

B

BLVRB

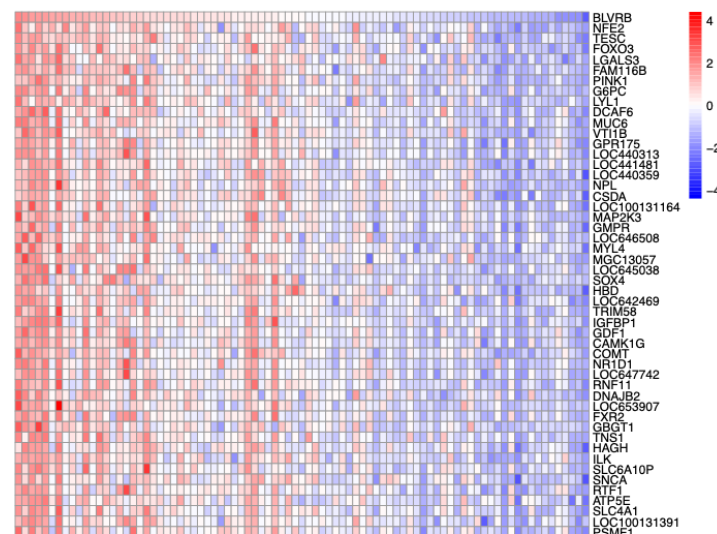

FIG

C

COX7C

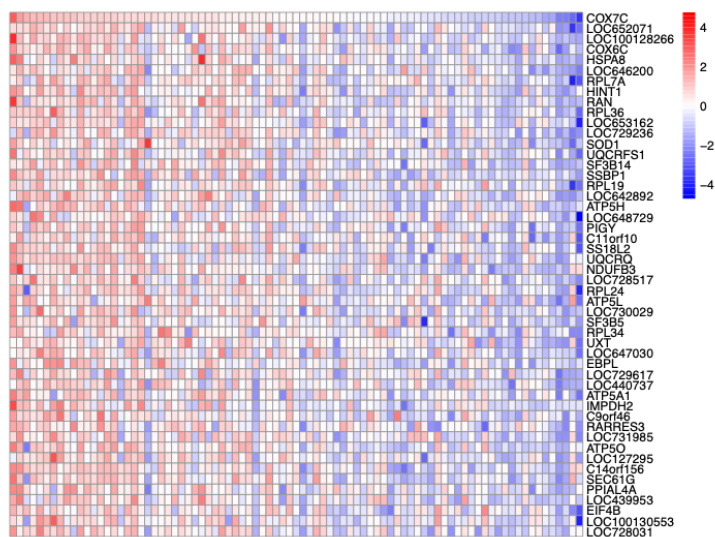

D

PIGY

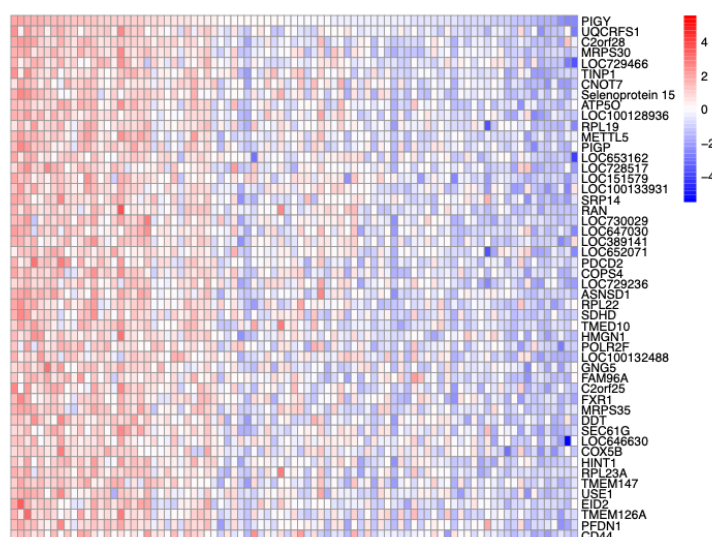

E

UOORQ

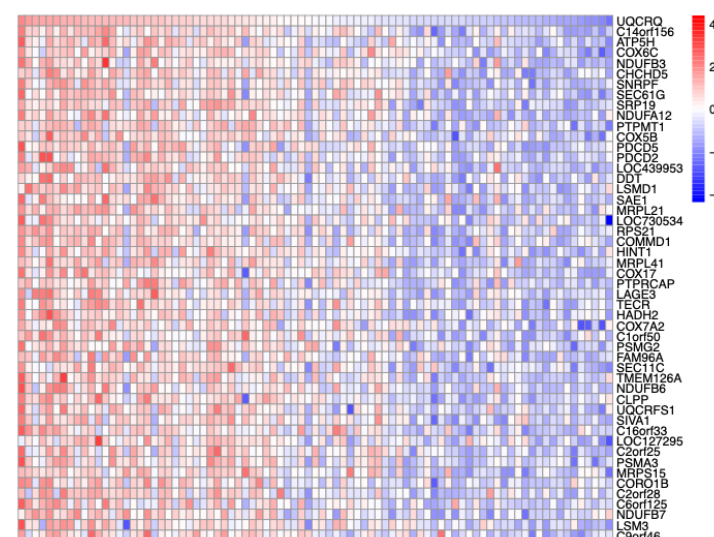

Supplement: Supplementary file 4 — Supporting Information [file CTM2-15-e70498-s006.pdf]

A

ATP5O

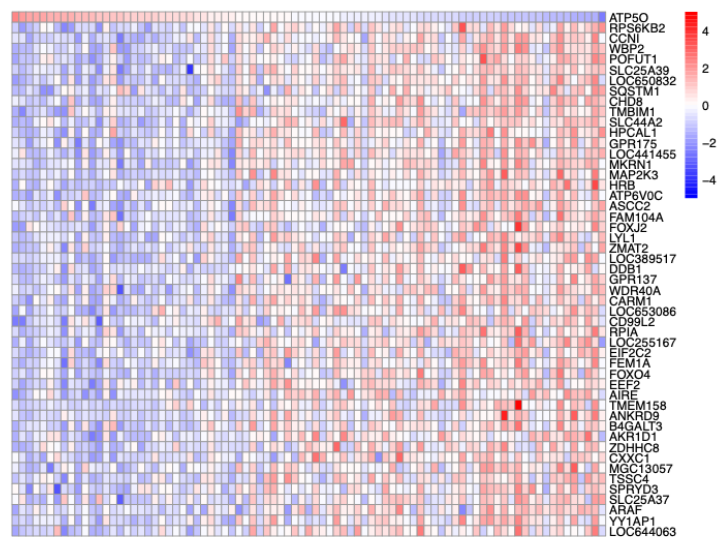

B

BLVRB

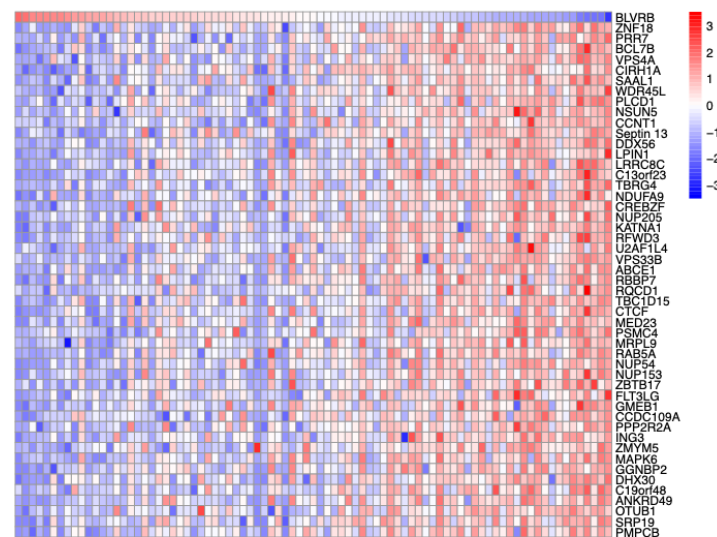

FIG

C

COX7C

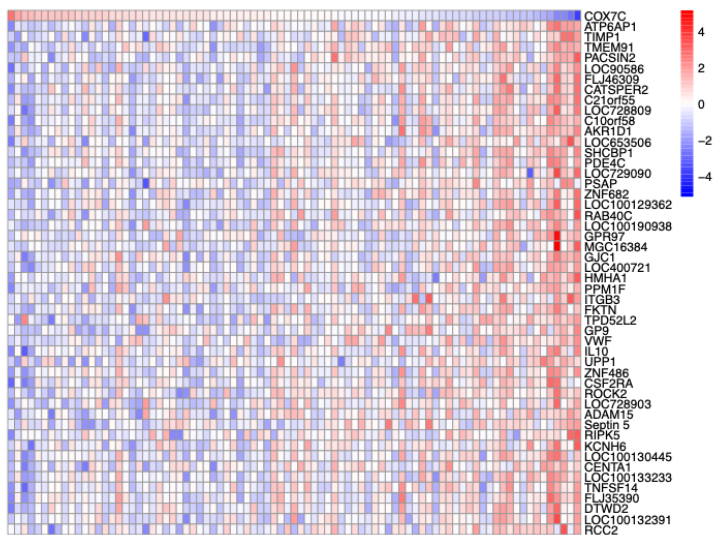

D

PIGY

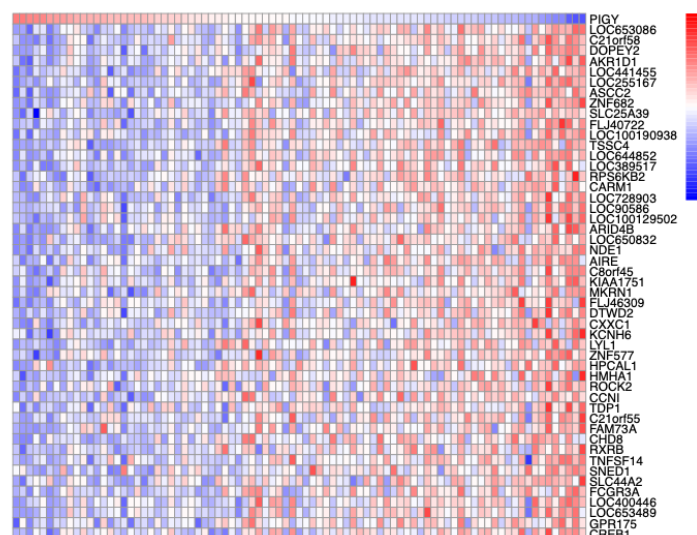

E

UQCRRQ

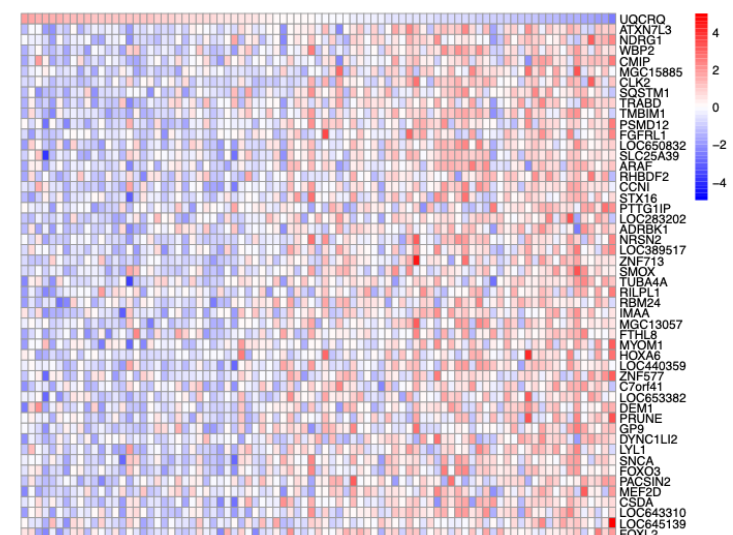

Supplement: Supplementary file 5 — Supporting Information [file CTM2-15-e70498-s003.pdf]

A

ATP5O

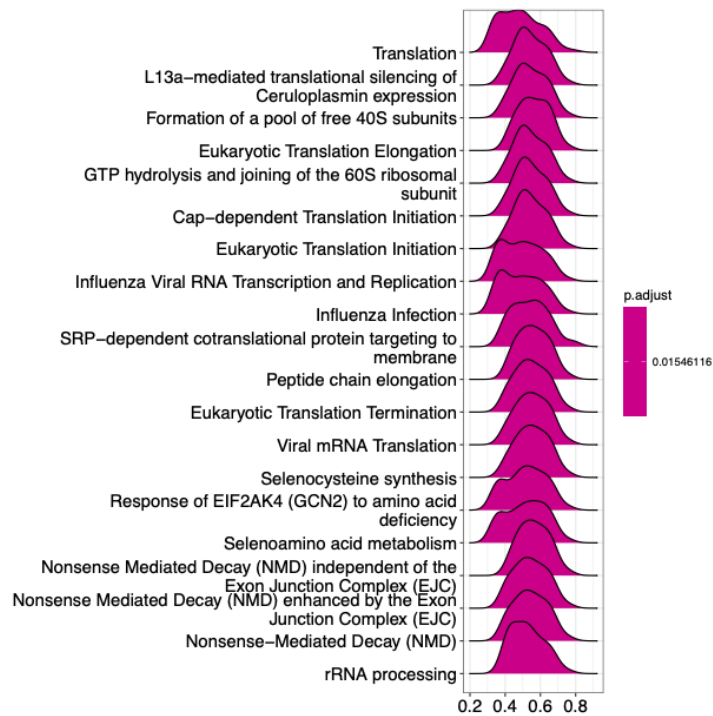

B

BLVRB

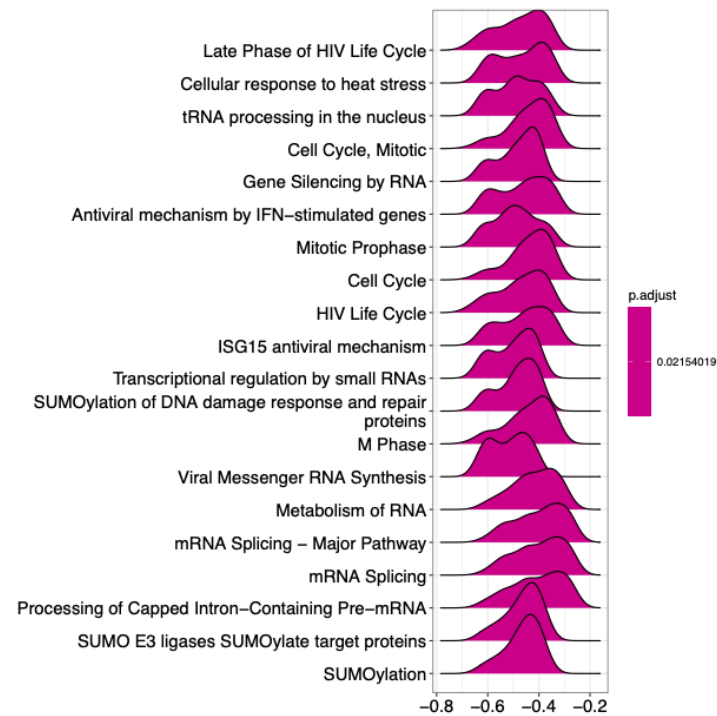

C

COX7C

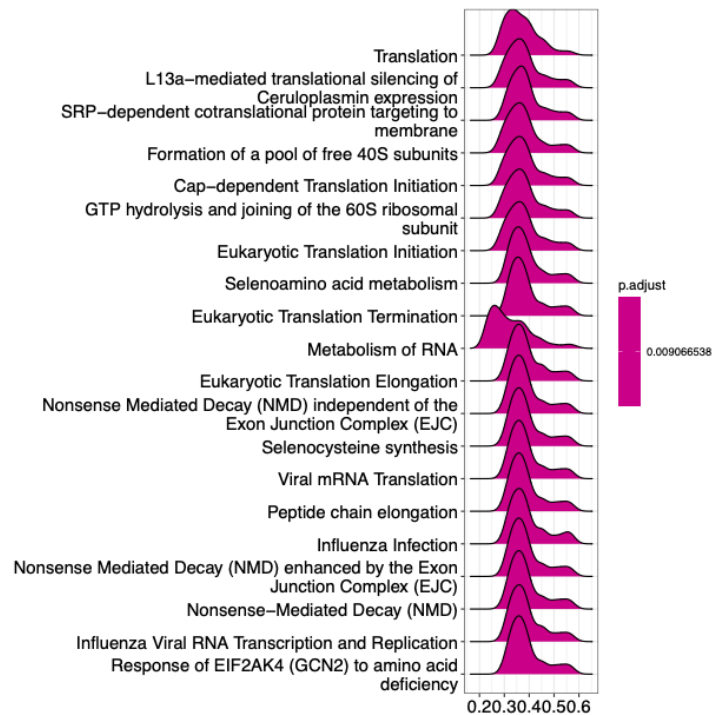

D

PIGY

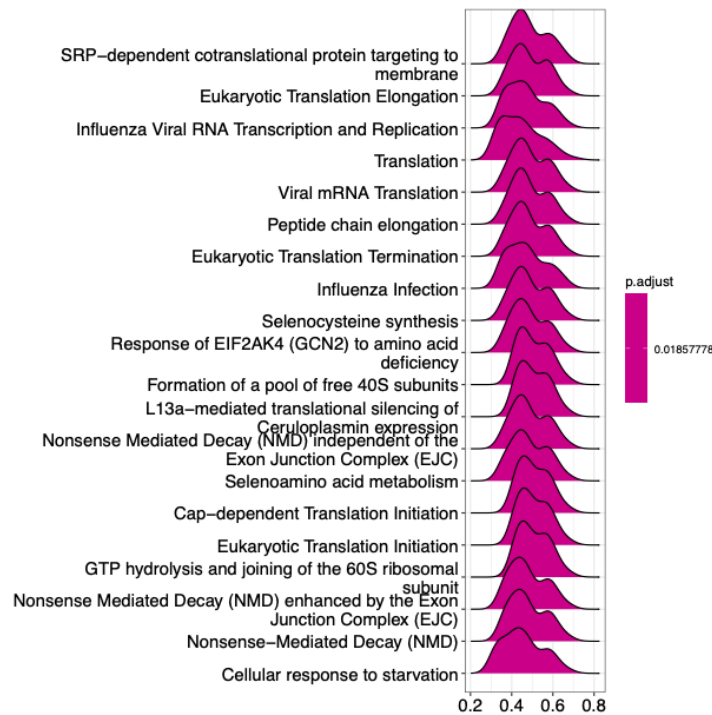

E

UQCRQ

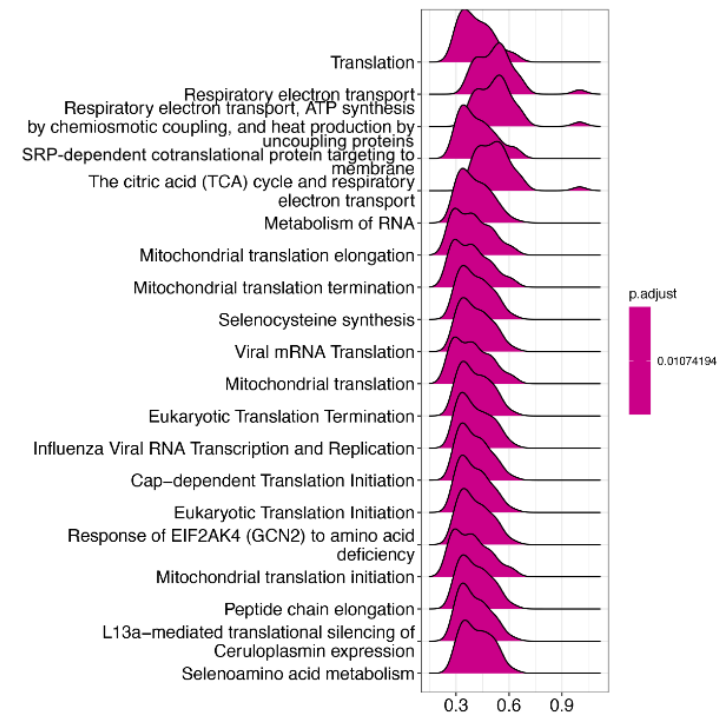

Supplement: Supplementary file 6 — Supporting Information [file CTM2-15-e70498-s007.pdf]

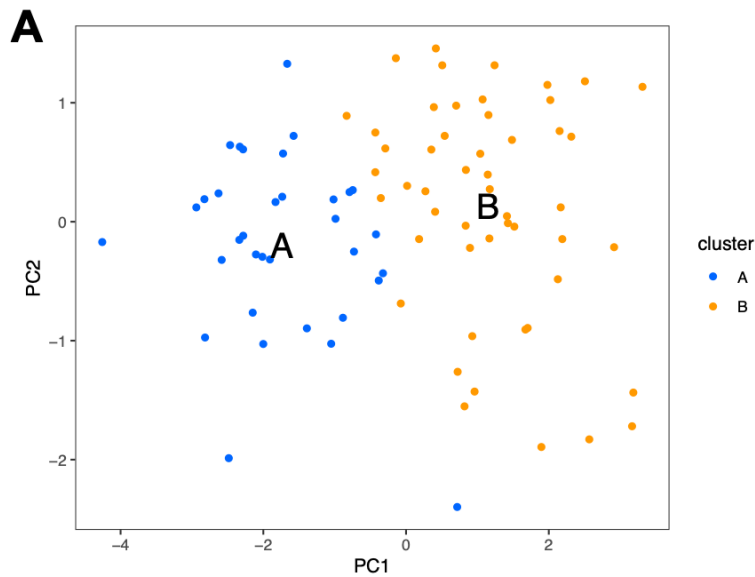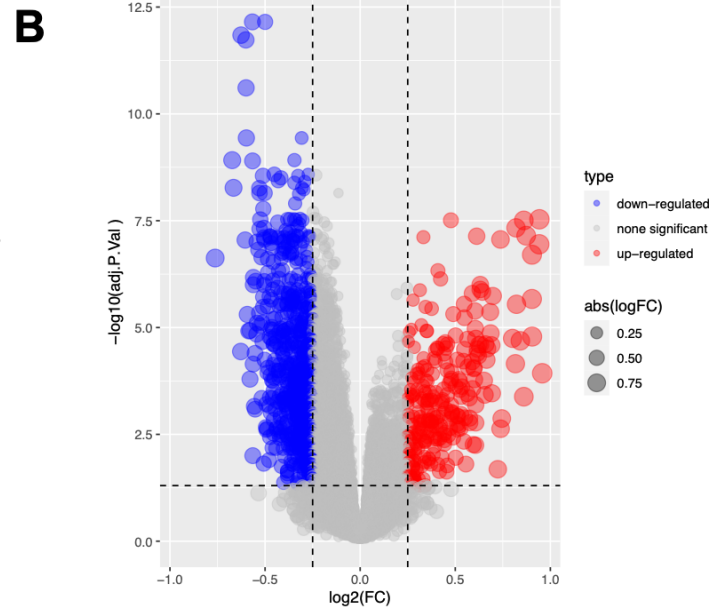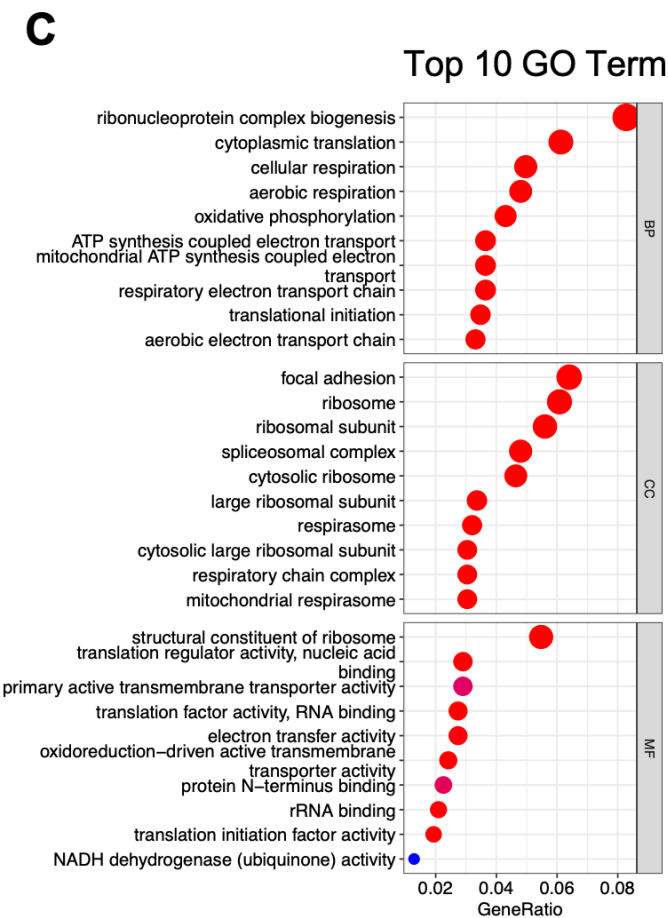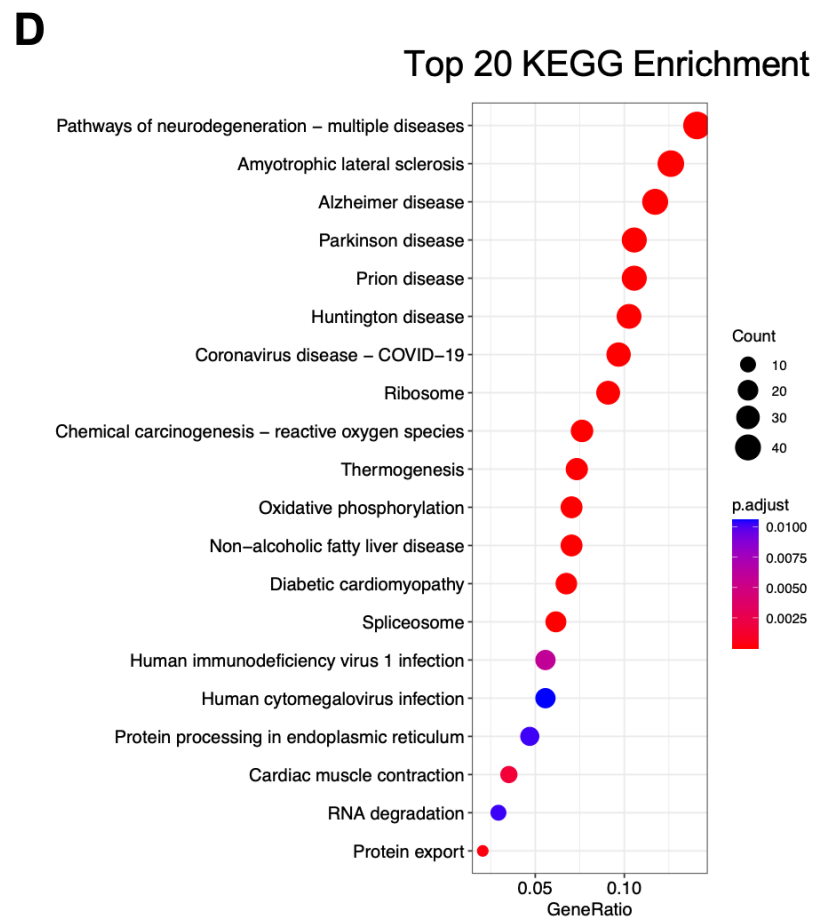

Supplement: Supplementary file 7 — Supporting Information [file CTM2-15-e70498-s009.pdf]
