# Supplementary material for: Immune cell subset profiling and metabolic dysregulation define the divergent immune microenvironments in HIV immunological non‐responders
Source: Clin Transl Med. 2025 Oct 13;15(10):e70498. doi: 10.1002/ctm2.70498 (PMC12518780; doi:10.1002/ctm2.70498)
Supplement: Supplementary file 8 — Supporting Information [file CTM2-15-e70498-s002.docx]

**Supplementary Table 1.** The classification and clinical information of enrolled 85 PLWH in GSE143742 and GSE106792 dataset.

|  | GSE143742 | GSE106792 |
| --- | --- | --- |
| INR group (n) | 44 | 12 |
| CD4 (cells/µL) | <350 | <350 |
| IR group (n) | 17 | 12 |
| CD4 (cells/µL) | >500 | >500 |
| ART-treated (Years) | >3 | 2-6 |
| Platform | GPL10558 Illumina HumanHT-12 V4.0 expression beadchip | GPL10558 Illumina HumanHT-12 V4.0 expression beadchip |
| Sample type | RNA | RNA |
| Tissue | Whole Blood | PBMC |
| Cell type | CD3^+^CD4^+^ T cells | CD3^+^CD4^+^CD45RA^–^CD71^–^ or CD71^+^ T cells |

PLWH, People living with HIV; INR, Immunological non-responder; IR, Immunological responder; ART, Antiretroviral therapy; PBMC, Peripheral blood mononuclear cell.
