# Supplementary material for: Immune cell subset profiling and metabolic dysregulation define the divergent immune microenvironments in HIV immunological non‐responders
Source: Clin Transl Med. 2025 Oct 13;15(10):e70498. doi: 10.1002/ctm2.70498 (PMC12518780; doi:10.1002/ctm2.70498)
Supplement: Supplementary file 9 — Supporting Information [file CTM2-15-e70498-s004.docx]

**Supplementary Table 2.** The information of primers sequences for qRT-PCR.

| Primer name | Sequence (5' to 3') | Base number |
| --- | --- | --- |
| ATP5O F | GACTGATCCGTCAATCTTGGG | 21 |
| ATP5O R | GACAATCTCCCGCATAGCCC | 20 |
| PIGY F | AGCCGCTCAGATATGAAGCAT | 21 |
| PIGY R | CATCCTAGCTGCCTGTGGTAT | 21 |
| UQCRQ F | ATCCGCACGTCTTCACTAAAG | 21 |
| UQCRQ R | TGGATCTCTCGAACTCTTCAGTC | 23 |
| COX7C F | GGTCCGTAGGAGCCACTATGA | 21 |
| COX7C R | GTGTCTTACTACAAGGAAGGGTG | 23 |
| BLVRB F | CAGGCTGTGACTGATGACCAC | 21 |
| BLVRB R | TCACTGTGTACGCCCCAGTTA | 21 |
| GAPDH F | GGAGCGAGATCCCTCCAAAAT | 21 |
| GAPDH R | GGCTGTTGTCATACTTCTCATGG | 23 |

F, Forward primer; R, Reverse primer.
