# Supplementary material for: Immune cell subset profiling and metabolic dysregulation define the divergent immune microenvironments in HIV immunological non‐responders
Source: Clin Transl Med. 2025 Oct 13;15(10):e70498. doi: 10.1002/ctm2.70498 (PMC12518780; doi:10.1002/ctm2.70498)
Supplement: Supplementary file 10 — Supporting Information [file CTM2-15-e70498-s008.docx]

**Supplementary Table 3.** 219 upregulated genes and 259 downregulated genes.

| Upregulated genes | Downregulated genes |
| --- | --- |
| FAM104A | ID3 |
| SLC25A39 | C2orf28 |
| CCNI | UQCRFS1 |
| RPS6KB2 | METTL5 |
| GPR175 | ATP5O |
| LOC440313 | MRPS30 |
| LOC644852 | ANKRD49 |
| LOC388621 | MRPS21 |
| HPCAL1 | SRP14 |
| WDR40A | PIGY |
| LOC642469 | TBCC |
| MAP2K3 | RBBP7 |
| TMBIM1 | PRPF31 |
| DNAJB2 | LOC729466 |
| RPIA | PSMG2 |
| MKRN1 | SRP19 |
| LOC441455 | ASNSD1 |
| LOC650832 | LOC728453 |
| THOC2 | PDCD6 |
| ZMAT2 | PFDN1 |
| RNF213 | DDT |
| WBP2 | ADRM1 |
| LOC643310 | RPL36 |
| LMAN1 | NUP153 |
| GNPNAT1 | UQCRQ |
| B4GALT3 | C7orf30 |
| LYL1 | C14orf156 |
| HBQ1 | SPAG7 |
| CARM1 | RPS21 |
| ALAS2 | LOC100128936 |
| ASCC2 | LOC644464 |
| HBG1 | DIMT1L |
| FLJ45337 | NGDN |
| ZBTB44 | CHMP1B |
| SPRYD3 | PPP3CC |
| LHPP | U1SNRNPBP |
| ATXN7L3 | MKI67IP |
| GATAD2A | POLR2F |
| SLC4A1 | GLRX |
| SRRD | NDUFA9 |
| EEF2 | APEX1 |
| LOC388532 | RPL19 |
| HBM | HINT1 |
| C7orf41 | RAB2A |
| ATP6V0C | PMPCB |
| BLVRB | COX5B |
| GMPR | ITGB4BP |
| GCC1 | C18orf21 |
| TCEB3 | MED30 |
| SNCA | GTF2B |
| KLHL22 | C12orf57 |
| AHSP | MOAP1 |
| SLC38A5 | CLNS1A |
| HCCA2 | ILF2 |
| LOC387686 | TMEM147 |
| FEM1A | LOC728517 |
| HBG2 | GLO1 |
| LOC654155 | LOC728031 |
| SPATA2L | LSMD1 |
| LOC731640 | LOC100133931 |
| WDR13 | IMPDH2 |
| FLJ44054 | RPL27A |
| IRX1 | TSEN54 |
| RNF11 | RPS9 |
| IFI27 | LOC100128266 |
| LOC646508 | JUNB |
| SLC25A23 | LOC644863 |
| RXRB | CPNE3 |
| EIF2C2 | LOC127295 |
| SESN3 | MGST3 |
| LOC643933 | TMED10P |
| ZNF428 | HMGN1 |
| LOC645284 | LOC100130553 |
| FXR2 | PHB2 |
| FOXJ2 | LOC728126 |
| PHLDB1 | LOC644988 |
| RAB35 | LOC730029 |
| TM7SF3 | DNAJB1 |
| TMOD1 | MRPL41 |
| LOC100131391 | SESN1 |
| C9orf78 | CCT8 |
| IL8RB | LOC651436 |
| SOX4 | STX8 |
| PSMF1 | TMEM85 |
| MOSPD3 | LOC151579 |
| FOXO4 | SEC61G |
| CYB5R3 | AP3B1 |
| YBX1 | MDH1 |
| LOC100190938 | TINP1 |
| UBE2F | BRI3 |
| TRIM58 | PPA1 |
| NUCB1 | RPL10A |
| LOC374395 | LOC441775 |
| MDH2 | RPS19BP1 |
| GPR137 | EIF2S3 |
| PP14571 | RPS5 |
| ZNF419 | IMP4 |
| LOC645038 | NDUFS5 |
| FAM116B | LOC441506 |
| LOC647742 | RPL24 |
| TUBA4A | CHURC1 |
| NR1D1 | C2orf25 |
| OR2W3 | MRLC2 |
| XPO7 | EIF4G2 |
| HBA1 | RPL23A |
| STK36 | IL1B |
| FOXO3 | SIVA1 |
| GPC6 | LOC341457 |
| HSPB1 | C5orf41 |
| CHST2 | IMP3 |
| CALM3 | EIF3D |
| GFOD2 | TOP2B |
| CAMK1G | VIM |
| RTF1 | MAPRE1 |
| TMEM158 | LOC100132742 |
| MAGEL2 | H2AFZ |
| MGC13057 | LOC645436 |
| PRR5 | LPIN1 |
| BCL2L1 | PEPD |
| GCN1L1 | AIP |
| CBX6 | FBXO33 |
| HBD | SRP14P1 |
| UROS | TMEM71 |
| TSSC4 | LOC646630 |
| CHD8 | LOC645296 |
| FAM83F | PCNP |
| LOC100134102 | LOC100131531 |
| GPR146 | C11orf10 |
| HIVEP3 | LOC730187 |
| C19orf22 | KLHDC2 |
| PHOSPHO1 | RPL22 |
| LOC389599 | SS18L2 |
| LOC100134364 | COX17 |
| SMOX | LOC644039 |
| GPA33 | C16orf33 |
| HAGH | NSA2 |
| SYT11 | LOC730246 |
| LOC338870 | SNORA24 |
| MIR1976 | MAP2K1IP1 |
| GLRX5 | LOC389672 |
| FIS1 | EIF4B |
| E2F2 | Selenoprotein 15 |
| LOC731777 | DDIT3 |
| CCDC51 | LOC387867 |
| RNF219 | ZNF148 |
| LOC100131164 | PROK2 |
| ACTRT1 | ADA |
| LOC440359 | TIGA1 |
| FCGR3A | COX7C |
| LOC100131205 | ATP5A1 |
| LGALS3 | ADPGK |
| LOC284422 | GPSM3 |
| LOC652140 | SIVA |
| LOC648526 | LOC643531 |
| GUK1 | LOC389342 |
| LOC100129502 | SGK |
| FBXO7 | FAM108A2 |
| STRADB | SFRS18 |
| LOC642333 | FLT3LG |
| GSPT1 | CLDND1 |
| VWCE | ANAPC5 |
| IGF2BP2 | COMMD3 |
| CTGLF7 | SNHG1 |
| TBC1D10B | DCTN5 |
| OSBP2 | C14orf166 |
| LOC643665 | LOC100132395 |
| CCDC52 | HSPA9 |
| KCNJ10 | ARPC3 |
| GANAB | LOC388654 |
| PIP5K2A | HBEGF |
| LOC730226 | LOC729236 |
| LOC645157 | LOC730534 |
| LOC646531 | LEF1 |
| CSDA | LOC728643 |
| DPYSL5 | RALB |
| VTI1B | SNRPB |
| TNS1 | RPS4X |
| SIAH2 | CD3G |
| LOC100132394 | LOC727865 |
| LOC642377 | LOC647030 |
| ATP5EP2 | NCRNA00219 |
| MED25 | SNORD68 |
| LOC653778 | RPL15 |
| TMEM86B | FLJ43681 |
| HBE1 | HNRNPL |
| LOC646463 | CCDC59 |
| HBB | HNRNPM |
| C22orf13 | PSMC2 |
| MYL4 | CYC1 |
| LOC100133551 | DDX39 |
| SELENBP1 | CALM2 |
| SLC25A37 | RPL17 |
| SERPINA13 | LOC647340 |
| STOM | SGK1 |
| EPB42 | PTPRCAP |
| HBA2 | SAT2 |
| CA1 | RPL13A |
| LOC653498 | LOC645385 |
| LOC648796 | SPCS1 |
| LOC100008589 | DDX18 |
| LOC100134053 | PRKCH |
| WAS | HSP90AB1 |
| SLC1A5 | RBM17 |
| C16orf35 | LOC729617 |
| TNIP1 | LOC647285 |
| UBXN6 | C9orf89 |
| IFIT1L | BTG1 |
| PINK1 | SETD2 |
| TSPAN5 | CXCR4 |
| PPP3R1 | CHES1 |
| RAB2B | ATP5C1 |
| LOC653635 | CD83 |
| FAM46C | PSMA5 |
| MFGE8 | LOC652624 |
| GP9 | HLA-DQA1 |
| LOC100129211 | SF3B5 |
| HPS1 | MED6 |
| UBE2M | LOC653881 |
| TMPRSS9 | NOSIP |
|  | C20orf111 |
|  | MAP3K8 |
|  | SFRS9 |
|  | TNF |
|  | LOC729301 |
|  | FNBP4 |
|  | PPP1R2 |
|  | CCDC12 |
|  | RNF103 |
|  | NELL2 |
|  | DHX15 |
|  | CCNL1 |
|  | BTG2 |
|  | LOC283412 |
|  | RBM5 |
|  | C9orf142 |
|  | RPL4 |
|  | LOC728835 |
|  | H3F3B |
|  | HNRNPH1 |
|  | LOC440063 |
|  | ALDH2 |
|  | ANXA1 |
|  | LOC731985 |
|  | SNORA70 |
|  | OCIAD2 |
|  | ID2 |
|  | LOC391833 |
|  | HSPA8 |
|  | CCDC130 |
|  | CLK1 |
|  | C12orf41 |
|  | LOC100131609 |
|  | YPEL5 |
|  | LSM7 |
|  | S100A4 |
|  | RP5-1022P6.2 |
|  | DDX5 |
|  | MYLIP |
|  | ATP6AP2 |
